# Effectiveness of digital physiotherapy interventions in patients with knee osteoarthritis: a systematic review and meta-analysis of randomised controlled trials

**DOI:** 10.1136/bmjopen-2025-102887

**Published:** 2025-12-11

**Authors:** Tsz Him Ryan Cheung, Mark T Elliott, Gareth Stephens, Michael Mansfield

**Affiliations:** 1 School of Sport, Exercise and Rehabilitation Sciences, College of Life and Environmental Sciences, University of Birmingham, Birmingham, UK; 2 The Royal Orthopaedic Hospital NHS Foundation Trust, Edgbaston, UK

**Keywords:** Knee, REHABILITATION MEDICINE, Telemedicine, Digital Technology

## Abstract

**Abstract:**

**Objectives:**

This systematic review and meta-analysis aims to provide an overview of the effectiveness of digital physiotherapy interventions on pain, physical functions and quality of life for patients with knee osteoarthritis.

**Design:**

Systematic review and meta-analysis using the Grading of Recommendation, Assessment, Development and Evaluation (GRADE) approach.

**Data sources:**

A systematic search of electronic databases, including MEDLINE, EMBASE, Web of Science, PsycInfo, CINAHL, Scopus and Cochrane Library, was conducted on 19 February 2025.

**Eligibility criteria for selecting studies:**

We included randomised controlled trials which compared digital physiotherapy interventions to standard physiotherapy care for patients with knee osteoarthritis. Main outcomes included pain, physical functions and quality of life.

**Data extraction and synthesis:**

25 studies met the inclusion criteria, and 18 studies were eligible for meta-analysis. The primary author conducted the initial search, selected articles and extracted data from eligible studies, which were independently checked by a second reviewer. Risk of bias (ROB) was assessed by Cochrane ROB-2 tool. Quality of evidence was evaluated by the GRADE approach.

**Results:**

Overall, digital physiotherapy was associated with a small but statistically significant improvement in physical function (SMD=0.24, 95% CI 0.13 to 0.35); an overall meta-analysis was not performed for pain and quality of life due to considerable heterogeneity. Subgroup analyses revealed both video-conferencing and app- or web-based physiotherapy significantly reduced pain (SMD=−0.53, 95% CI −1.06 to −0.01 and SMD=−0.47, 95% CI –0.70 to −0.25, respectively) and physical function (SMD=0.32, 95% CI 0.10 to 0.54 and SMD=0.30, 95% CI 0.09 to 0.50 respectively). Digital physiotherapy interventions with individualised exercise components also reduced pain (SMD=−0.43, 95% CI −0.66 to −0.21) and improved physical function (SMD=0.30, 95% CI 0.17 to 0.43), when compared with non-exercise interventions.

**Conclusion:**

There was moderate-quality evidence to support the use of digital physiotherapy interventions in improving pain and function in patients with knee osteoarthritis. Subgroup analyses revealed low-to-moderate quality evidence in using video-conferencing and app-/web-based physiotherapy and interventions with exercise components to treat patients with knee osteoarthritis. Overall, there were limited high-quality trials in drawing a robust conclusion. High ROB and huge heterogeneity were observed across studies. Further research should minimise the ROB and investigate the effect of different digital modalities, intervention components and length of follow-up.

**STRENGTHS AND LIMITATIONS OF THIS STUDY**

- This study adhered well to the guidance of PRISMA recommendations and thoroughly assessed risk of bias and the quality of evidence across all included studies.
- The review included an ample amount of studies (25 randomised controlled trials)—21 of them were published after 2020, offering a contemporary perspective of the topic.
- Study selection and data extraction were completed by the primary author and only independently checked by a second reviewer, which could have led to potential selection bias.
- High risk of bias, mainly due to lack of blinding and high attrition rate, was observed in nearly half (11 out of 25) of the included trials.
- Substantial heterogeneity was observed across studies, which could have impacted the clinical significance of the results.

## Introduction

 Osteoarthritis (OA) is a degenerative joint disease which damages the articular cartilage, leading to pain and functional disability.^1^ It is one of the leading contributors to disability worldwide^2^ and has seen the largest rise in prevalence of 114% since 1990 compared with other musculoskeletal conditions.^3^ Knee OA is the most common form of OA,^4^ and a high prevalence of 2.9% was recorded in the United Kingdom in 2017.^5^ Knee OA significantly hinders physical function and activities of daily living.^1^ Owing to its persistent nature, management of knee OA can result in significant cost to healthcare services.^6^ On average, a single total knee replacement costs £7500 including post-operative care,^7^ with around 75 000 knee replacement procedures performed each year in the UK (NHS, 2021–2022^8^). Given the substantial economic implication of surgical management, there has been growing emphasis on conservative management to potentially delay or avoid the need for surgery.^4^

Core elements of physiotherapy, including exercise therapy and education, are recommended by National Institute for Health and Care Excellence (NICE) guideline as first-line treatment for knee OA.^9^ However, lack of time, accessibility and low self-regulation were some major barriers preventing patients from adhering to treatment.^10^ In light of this, digital health interventions have been evolving as an adjunct for patients with knee OA.^11^ According to the WHO, a digital health intervention is defined as a “discrete functionality of digital technology that is applied to achieve health objectives and is implemented within digital health applications and ICT systems, including communication channels such as text messages”.^12^ In the context of physiotherapy, phone consultation, telehealth practice and app-based exercise prescription were some of the most common digital health interventions.^13^

Having the potential to enhance cost-effectiveness in healthcare,^14^ there has been a growing body of literature in digital physiotherapy interventions for patients with knee OA. However, findings into clinical outcomes of digital physiotherapy have been inconsistent. A number of meta-analyses showed that telerehabilitation only improved pain, but not physical function in patients with knee OA.^15^,^17^ Conversely, telerehabilitation, digital self-management programmes and electronic-based home exercise interventions were reported to show significant improvement for people with knee OA in both pain and physical function, some of which could be maintained at 12 month follow-up.^18^,^20^ A more recent meta-analysis of 13 studies also showed that digitally delivered exercise was significantly more effective than education and paper-based delivery exercises in improving quality of life (QoL) in people with knee OA, but it was only comparable to face-to-face delivery for pain, function and QoL.^21^ Moreover, most studies focused on restricted options of digital medium and did not consider a wider spectrum of delivery modes, such as digital gaming and text messages. There has also been little agreement from recent reviews on the effect of the types of content delivered via digital interventions,^18 22^ as well as the length of intervention follow-up.^18 19^ Therefore, this study seeks to analyse the effect of digital physiotherapy interventions on pain, physical functions and QoL in patients with knee OA, addressing the following research questions: (1) Are digital physiotherapy interventions associated with better, or non-inferior pain, physical function and QoL, when compared with physiotherapy care with no or insignificant digital components? (2) Which digital medium or/and component of intervention yields the best clinical outcomes? (3) How long can these improvements, if any, be maintained?

## Methods

This review was conducted in accordance with the guidance of Preferred Reporting Items for Systematic Reviews and Meta-Analyses (PRISMA) recommendations.^23^ PROSPERO Registration: CRD420250600819.

### Information Sources and Search Strategy

Studies were identified in seven databases, including MEDLINE, EMBASE, Web of Science, PsycInfo, CINAHL, Scopus and Cochrane Library, by the primary author. Both Medical Subject Headings terms and free-text words were used to search for randomised controlled trials (RCTs) relevant to digital physiotherapy interventions and knee OA published from January 1, 2013, to February 19, 2025. The date last searched was February 19, 2025. The review was originally designed and conducted as part of a project in 2023, with an intended 10-year search window (January 2013 – December 2023). Before submission for publication, the search was updated in February 2025 to capture newly published trials and maintain the review’s currency. This resulted in an extended range from 2013 to 2025. Non-English studies were excluded because validated translation services were not available within the project resources. Detailed search strategies for all databases are described in online supplemental appendix I.

### Eligibility criteria

#### Participants

Adults who were 18 years old or above with a diagnosis of knee OA were included. Diagnostic criteria included any one of the following: (1) NICE guideline criteria,^9^ (2) American College of Rheumatology criteria,^24^ (3) radiologic evidence of OA according to Kellgren and Lawrence score, grade two or above,^25^ or (4) a reported diagnosis by physicians. All stages of OA were considered. Participants who had, or were on a waiting list for knee surgery, were excluded.

#### Intervention groups

Interventions should at least include one component of physiotherapy that was delivered through means of digital technologies. Referring to the taxonomy of digital health by WHO, this review would focus on two domains: (1) Client-to-Provider Telemedicine, defined as “provision of health services at a distance” and (2) Targeted Client Communication, defined as “transmission of customised health information for different audience segments”.^12^ These could include but are not limited to telephone calls, text messages, video conferencing, web- or app-based programmes and digital video games. Components of physiotherapy would adhere to the recommendations by NICE guidelines, including any one of the following: (1) Information, education and support, (2) Therapeutic exercise, (3) Weight management and (4) Manual therapy alongside therapeutic exercise.^9^

#### Comparison groups

Standard physiotherapy care (ie, any one of the components listed above from (1) to (4) in the NICE guideline) with no or insignificant components of digital health would be considered. The use of digital physiotherapy care that was not included in its corresponding intervention group was also included. Comparison groups that included passive care, such as being on waiting lists, or medical care, such as medication or surgery, were excluded from this review.

### Outcome measures

The primary outcomes for this review included pain and physical function. Secondary outcome was QoL. The following outcome measures or subscales were included:

1. Pain: Numerical Rating Scale (NRS), Visual Analogue Scale (VAS), pain subscale in The Western Ontario and McMaster Universities Osteoarthritis Index (WOMAC) and pain subscale in Knee Injury and Osteoarthritis Outcome Score (KOOS)
2. Physical functions: Physical function subscale in WOMAC and activities of daily living subscale in KOOS
3. Quality of life: Assessment of Quality of Life (AQoL), WHO Quality of Life – Brief Vision (WHOQOL-BREF), Short Form of Health Survey Questionnaire (SF-36), Arthritis Research UK Musculoskeletal Health Questionnaire (MSK-HQ), KOOS subscale in knee-related QoL and WOMAC total score, European Quality of Life 5 Dimensions 5 Level Version (EQ-5D-5L)

### Types of studies

RCTs published in the English language were included. Since technology has been advancing rapidly in recent years, some forms of digital health, such as DVDs, were less available and relevant.^26^ Therefore, only studies published in the most recent ten years would be included.

### Study selection

The primary author screened the titles and abstracts of the search results against the eligibility criteria. For each selected study, a full text was retrieved. A second reviewer checked the selected papers against the inclusion and exclusion criteria independently from the primary author. Should any disagreement arise, discussion would be initiated to resolve the issues to minimise bias.

### Data extraction

Data was extracted by the primary author initially. The second reviewer checked the data against all the included papers independently from the primary author. Should any disagreement arise, discussion would be initiated to resolve the issues to minimise bias. The following data were extracted from all the selected studies: author, year of publication, country, sample size, mean age and percentage of male / female participants, types of digital medium used, treatment content and duration of the interventions, comparison groups, length of follow-up and outcome measures used. For each outcome measure, mean differences, standard deviations (SDs), 95% CI and p-values were extracted.

### Missing information and data

Should there be any missing information or data, corresponding authors of the papers would be contacted. One author was contacted to clarify the details of a control intervention during full-text screening, and the paper was excluded after clarification.^27^

### Risk of bias (ROB) and certainty assessment

The Cochrane ROB-2 Tool was used to assess the overall ROB of individual studies^28^: Studies were rated “low risk of bias” if all five domains were judged “low risk”. Studies were rated “some concerns” if at least one domain raised “some concerns” but none were “high risk”. Studies were rated “high risk of bias” if any single domain was judged “high risk” or if multiple domains raised “some concerns”. This overall judgement was used to conduct the sensitivity analysis, where studies rated “high risk of bias” were excluded to test the robustness of pooled results. ROB was assessed for each primary outcome, respectively, as these were included in quantitative synthesis. For studies reporting multiple outcomes, ROB for each of these outcomes was assessed separately according to the five ROB-2 domains. The primary author assessed the ROB independently, and the second reviewer reviewed and checked the results against the ROB criteria. The Microsoft Excel tool from ROB-2 Tool was used for automation purposes. All studies were evaluated against five domains of ROB: randomisation process, deviations from the intended interventions, missing outcome of data, measurement of the outcome and selection of the reported result.

To assess the certainty of the studies, the Grading of Recommendations Assessment, Development and Evaluation Assessment (GRADE) was used to address study limitations, imprecision, inconsistency of results, indirectness of evidence and publication bias.^29^ The GRADE approach was applied to maximally seven most important patient-reported comparisons, as recommended by the Cochrane Handbook to inform decision-making processes.^30^ Quality of synthesised evidence was rated as high, moderate, low or very low according to GRADE criteria.^29^ The Robvis tool was used to generate the ROB figure.^31^ The GRADEPro GDT online tool was used to generate the summary of finding table.^32^

### Statistical analysis

Cochrane RevMan 5.4 was used to conduct meta-analysis to pool the data of all eligible RCTs.^33^ Standardised mean difference (SMD) was used as the outcome measure because the included studies used different scales to evaluate the same outcome measure. The SDs of within-group mean differences in each study were used to calculate the SMDs by RevMan 5.4. Before conducting meta-analysis, heterogeneity across studies was assessed by Chi square test and *I*^*2*^ statistics. When moderate to high heterogeneity was observed among studies (p<0.05 or *I*^*2*^>50%), a random-effects model was used to synthesise the data; otherwise, a fixed-effects model was used.^34^ If considerable heterogeneity was observed (*I*^*2*^>75%), meta-analysis was not conducted.^34^ Subgroup analyses were performed to investigate the effect of: (1) different digital modalities, (2) components of specific individualised exercise and (3) length of follow-up on all outcome measures. To ensure the robustness of evidence, sensitivity analyses were performed to evaluate the effects of digital interventions by excluding studies with a high ROB.

### Patient and Public Involvement and Engagement (PPIE) Statement

Patients and members of the public were not involved in the design, conduct, or analysis of this systematic review. However, the findings will be shared with relevant patient and public groups to inform future research and dissemination strategies.

## Results

### Study selection

**Figure 1:**
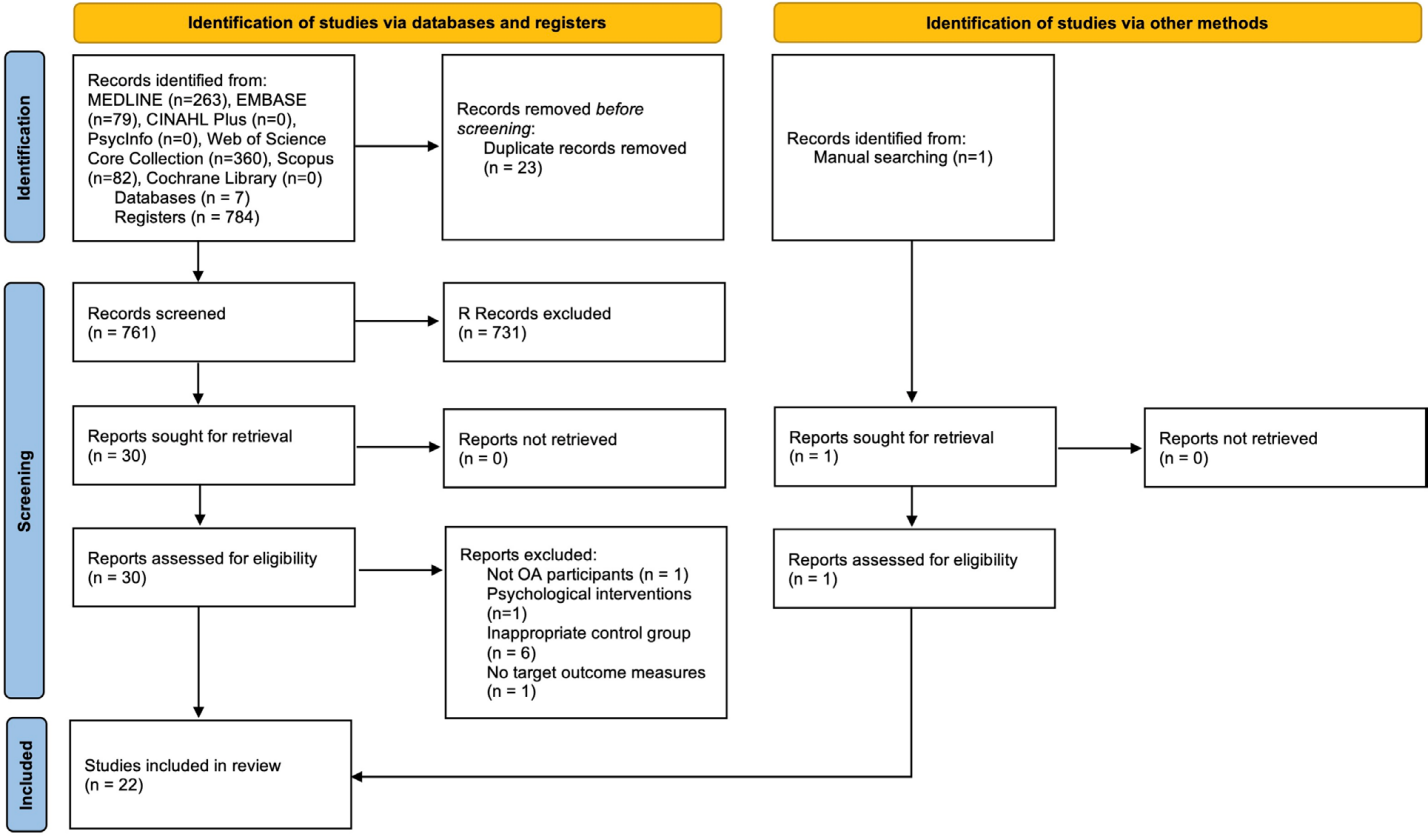
Preferred Reporting Items for Systematic Reviews and Meta-Analyses (PRISMA) Flow Diagram. OA, osteoarthritis.

A PRISMA flow chart indicating the selection process of studies is shown in figure 1. 1376 potential citations were identified (290 from MEDLINE, 448 from EMBASE, 532 from Web of Science and 99 from Scopus) in the initial search, and one citation was obtained from manual search. Upon title and abstract screening by the primary author, 1304 articles did not meet the inclusion criteria. Following full-text screening of 42 articles, 25 articles published between 2017 and 2024 were included, with a total of 3436 participants. No unresolved disagreements occurred between reviewers. The detailed reasons for exclusion are presented in online supplemental appendix I.

### Missing data

15 papers were identified as having missing data (SDs of within-group mean differences), and their corresponding authors were contacted by email. One author provided the missing data for two studies,^35^,^37^ while the missing data could not be retrieved from the remaining 13 studies. The last day to retrieve missing data was 5 March 2025. Missing within-group mean differences and SDs were calculated by using 95% CI or other relevant data in Cochrane RevMan 5.4.^33^

### Study characteristics

Study characteristics of all 25 studies are detailed in table 1 in online supplemental appendix II. No unresolved disagreements occurred between reviewers during data extraction. All participants were diagnosed with knee OA with either one of the four diagnostic criteria mentioned in the Methods section. The mean age (SD) of participants ranged from 51.73 (3.96) to 76 (16.9). Two studies^37 38^ only included female participants, while all other studies included both male and female. Detailed study-level findings for each respective outcome measure are presented in online supplemental appendix III.

### Intervention groups

According to WHO definitions,^12^ 21 out of 25 studies utilised digital health in the category “client-to-provider telemedicine”^35^,^55^ and four studies were categorised into “targeted client communication”.^36^,^59^ In terms of digital interventions, three studies used telephone to deliver coaching, counselling or exercise advice,^39 41 42^ four studies used text messages to send reminders to participants,^56^,^59^ seven studies used video-conferencing to deliver exercise programmes, monitor participants’ progress or provide education,^40 44 48 50 51 53 55^ eight studies used mobile applications or web pages to deliver exercise programmes and/or education,^35^,^3745 46 49 54^ and three studies incorporated exercises into digital video games.^43 47 52^ Length of follow-up ranged from 2 weeks to 24 months.

### Comparison groups

15 studies included conventional face-to-face physiotherapy,^3537 39^,^41 43 47 48 51^ four studies used paper exercise handouts^38 49 50 54^ and six studies provided general information on OA, exercise and/or self-management via mail, in-person sessions or internet.^3642 44^,^46 58^ One study also incorporated electrotherapy into both experimental and control groups in addition to exercise programmes.^52^

### Risk of bias assessment

**Figure 2:**
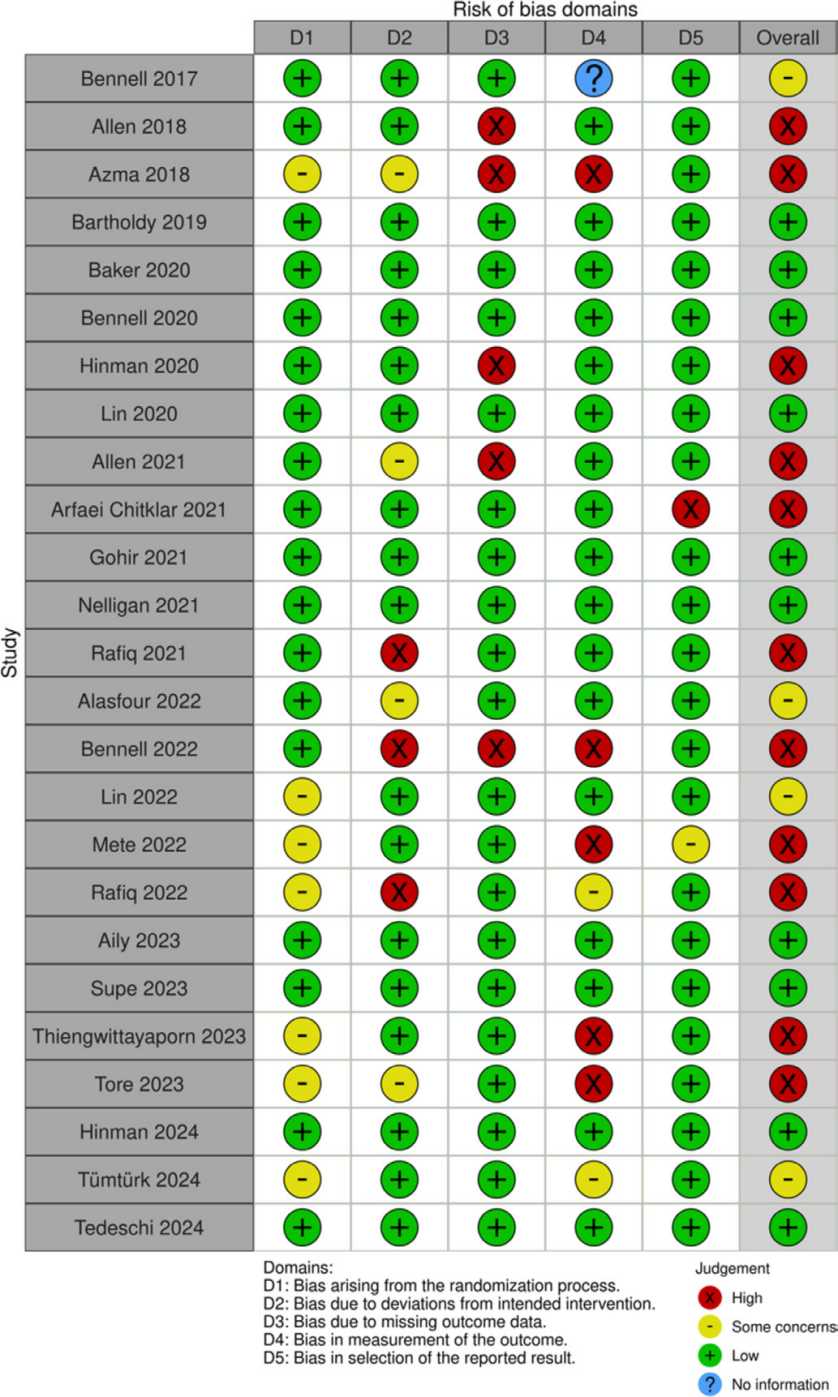
Risk of Bias summary for 25 RCTs. RCTs, randomised controlled trials.

The ROB results are shown in figure 2. No unresolved disagreements occurred between reviewers during ROB assessment. Overall, 11 studies had high ROB,^35^,^3740 42 44 49 50 52 58 59^ while 10 studies had low ROB.^4143 45 46 48 51 53 55^,^58^ The absence of blinding on participants was the most common item, leading to outcome assessment bias in eight studies.^39 40 44 49 50 52 54 59^ Four studies^4245^,^47 53^ used the principle of “limited disclosure”, which blinded participants to trial hypotheses and purposes, potentially reducing assessment bias.^60^ Five studies had high attrition rates (<85% retention)^35 36 40 42 44^ without appropriate reasons, leading to attrition bias.^61^ Seven studies did not present information on allocation concealment, thus selection bias was in question.^40 47 49 50 52 54 59^

### Meta-Analysis

#### Physical functions

**Figure 3:**
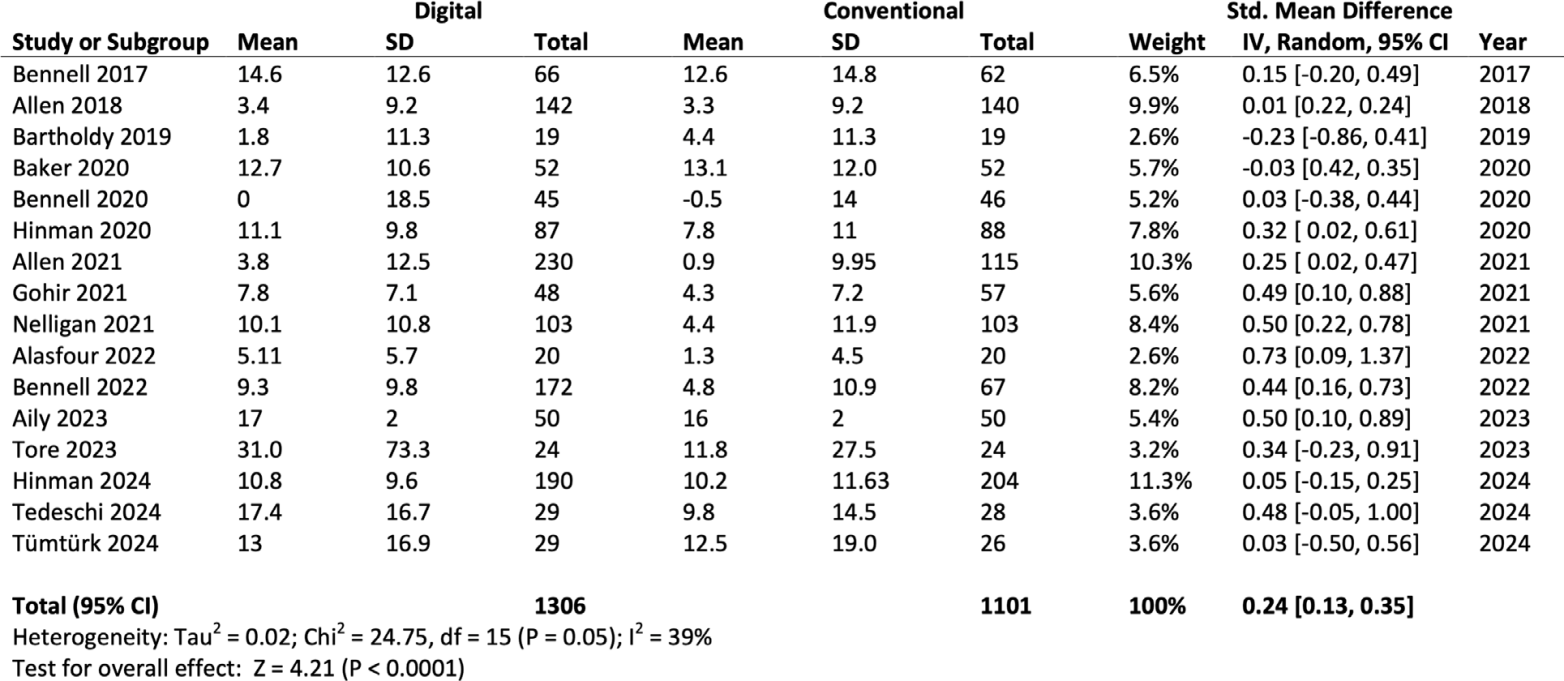
Results of meta-analysis of overall physical function of 16 RCTs. RCTs, randomised controlled trials.

**Figure 4:**
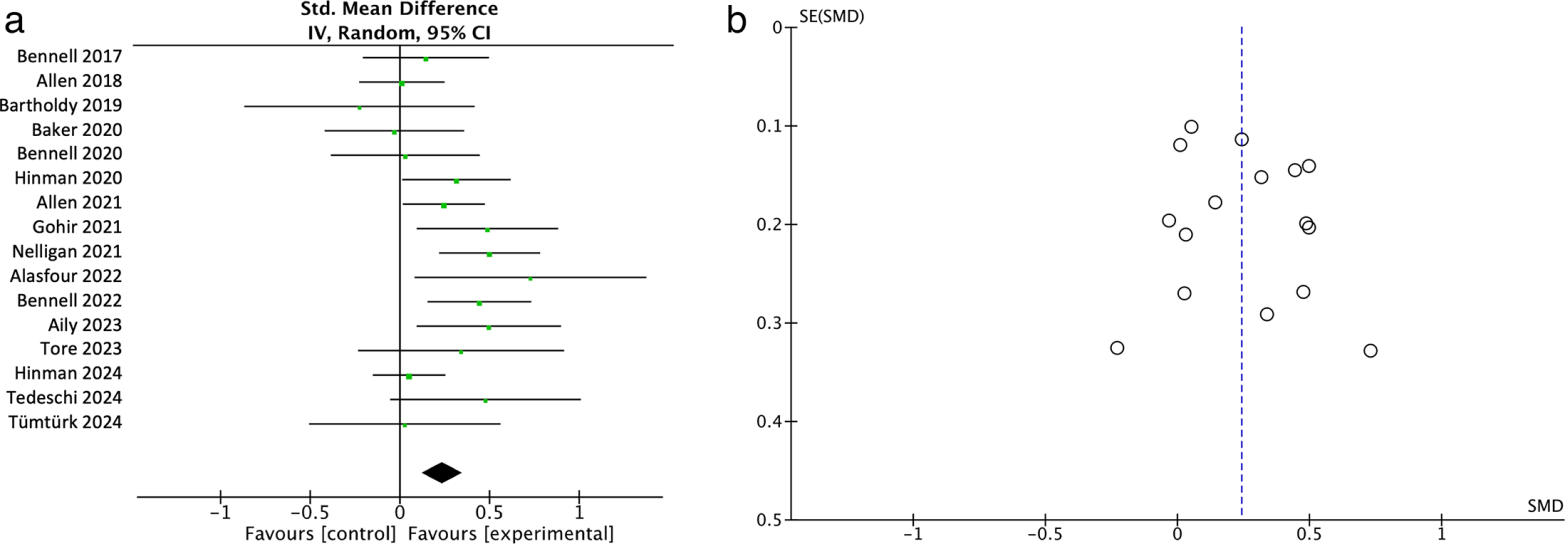
(a) Forest Plot of the meta-analysis of physical function on the above 16 RCTs. (b) Funnel plot for 16 RCTs included in meta-analysis for physical function. RCTs, randomised controlled trials.

16 studies with 2407 participants evaluated the effects of digital physiotherapy on physical functions compared with standard physiotherapy care. A significant result and small effect size was observed in favour of digital physiotherapy interventions (SMD=0.24, 95% CI 0.13 to 0.35; figure 3), rated with moderate-quality evidence using the GRADE approach.^29^ A moderate heterogeneity (*I*^*2*^=39%) was noted, indicating some variability in the true effect size across populations and intervention types. Length of follow-up ranged from 6 weeks to 24 months. The GRADE summary of findings table is shown in online supplemental appendix IV and figure 4a presents the forest plot of the results of the above analysis. The funnel plot is presented in figure 4b. Although the funnel plot for physical function outcomes appeared symmetrical, suggesting limited evidence of publication bias,^60^ the presence of small-study effects cannot be completely excluded. Several included trials had relatively small sample sizes and reported positive findings, which may overestimate treatment effects. Furthermore, studies with null or unfavourable results may be less likely to be published, contributing to potential publication bias. These factors should be considered when interpreting the pooled estimates, particularly given the heterogeneity observed across digital intervention types and study designs.

#### Pain

Due to considerable heterogeneity observed (p<0.001, *I*^*2*^=77%), an overall meta-analysis was not indicated for pain. Heterogeneity was mainly attributable to differences in delivery format, intervention content and length of follow-up. Overall, qualitative synthesis of 24 RCTs with 3398 participants showed digital physiotherapy was more effective, or at least non-inferior to conventional physiotherapy or home exercise programmes with paper handouts in alleviating pain. Nine RCTs^36^,^3844^ utilising video-conferencing and app-/web-based physiotherapy showed statistically significant pain improvement compared with non-digital interventions. However, the three most recent RCTs published in 2024 concluded no significant differences in pain relief between tele-rehabilitation and conventional physiotherapy.^53^,^55^ Four of the studies using telephone-based and text-based interventions did not show any additional benefits.^39 41 55 56^ Mixed results were found for digital video gaming. One trial^43^ using active video games (playing whack-a-mole and archery) showed no significant pain reduction, while another study using exergaming^52^ demonstrated effective pain relief when compared with conventional exercises.

#### Quality of life

Due to considerable heterogeneity observed (p<0.001, *I*^*2*^=81%), an overall meta-analysis was not indicated for QoL. The observed heterogeneity was largely driven by variations in delivery format, use of different outcome measures and length of follow-up. Overall, qualitative synthesis of 15 RCTs with 1601 participants demonstrated contradictory results for QoL improvement. Eight studies showed significant improvement,^37 39 43 44 46 49 50 54^ while seven studies did not suggest any benefits for digital physiotherapy compared with standard physiotherapy.^39 42 45 53 54 56 57^

### Subgroup analysis

Forest plots of all subgroup analyses are presented in online supplemental appendix V.

#### By digital medium

The types of interventions used by the included studies were divided into five groups (see Intervention Groups section): 1). Telephone-based, 2). Text Message-based, 3). Digital video game, 4). Video-conferencing and 5). App-/Web-based. Due to insufficient data, studies of digital video games were not included in meta-analysis. Planned subgroup analysis revealed that physiotherapy through video-conferencing showed small-to-moderate effect size in improving physical function (SMD=0.32, 95% CI 0.10 to 0.54), with low-quality evidence. App-based or web-based interventions showed small effect size (SMD=0.30, 95% CI 0.09 to 0.50), with moderate-quality evidence. Between-study heterogeneity was moderate (I² = 39%), indicating some variability in the true effect size across populations and intervention types. For pain, video-conferencing physiotherapy yielded the highest effect in pain reduction (SMD=−0.53, 95% CI −1.06 to −1.01), with low-quality evidence. This was followed by app-/web-based interventions (SMD=−0.47, 95% CI −0.70 to −0.25), with moderate-quality evidence. Between-study heterogeneity was high (I² = 77%), indicating large variability in the true effect size across populations and intervention types. For QoL, only three studies in app-based/web-based interventions revealed significant results with small-to-moderate effect size (SMD=0.42, 95% CI 0.04 to 0.80). Between-study heterogeneity was high (I² = 81%), indicating large variability in the true effect size across populations and intervention types. Insignificant results were obtained for telephone-based and text message-based interventions across all three outcome measures. Subgroup differences were statistically not significant across all three outcome measures (p=0.11, *I*^*2*^=50.9%; p=0.29, *I*^*2*^=19.8%; p=0.27, *I*^*2*^=23.4%, respectively). Therefore, the results should be interpreted with caution.

#### By component of individualised exercise advice/programme

All studies were divided into: (1) interventions with components of individualised exercise advice/programme and (2) interventions without individualised exercise components. Digital physiotherapy with individualised exercise components had significant improvement in physical function, with a small effect size (SMD=0.30, 95% CI 0.17 to 0.43), with moderate-quality evidence. Subgroup differences were significant (p=0.03, *I*^*2*^=79.2%). Between-study heterogeneity was moderate (*I²* = 39%), indicating some variability in the true effect size across populations and intervention types. Pain was also significantly improved with small-to-moderate effect size (SMD=−0.43, 95% CI −0.66 to −0.21), with moderate-quality evidence. Subgroup differences were not significant (p=0.15, *I*^*2*^=52.9%). Between-study heterogeneity was high (*I²* = 77%), indicating large variability in the true effect size across populations and intervention types. A significant effect on QoL was observed with a small effect size (SMD=0.21, 95% CI 0.03 to 0.40). However, subgroup differences were also not significant (p=0.61, *I*^*2*^=0%). Between-study heterogeneity was high (*I²* = 81%), indicating large variability in the true effect size across populations and intervention types.

#### By length of follow-up

Based on previous reviews, all studies were divided into: (1) 3 months or less, (2) 3–12 months and (3) 12 months or longer.^19 62^ Subgroup analysis showed that length of follow-up of 3 months or less yielded significant improvement in physical function with the largest effect size (SMD=0.33, 95% CI 0.05 to 0.60), with decreasing effect size as length of follow-up increased. However, while the point estimates varied, the 95% CI of individual subgroups overlapped. Therefore, no subgroup differences were observed (p=0.75, *I*^*2*^=0%). Between-study heterogeneity was moderate (*I²* = 39%), indicating some variability in the true effect size across populations and intervention types. Pain also followed the same trend, with the largest effect size noted in the subgroup of 3 months or less (SMD=−1.08, 95% CI −1.84 to −0.32), while the subgroup of 12 months or longer had the smallest effect size (SMD=−0.18, 95% CI −0.32 to −0.03). Subgroup differences were also not significant (p=0.07, *I*^*2*^=62.9%). Between-study heterogeneity was high (*I²* = 77%), indicating large variability in the true effect size across populations and intervention types. Results were not significant for all subgroups for QoL.

### Sensitivity analysis – ROB assessment

Sensitivity analysis was performed for the outcome of physical functions with the exclusion of five studies with high ROB. Significant results and similar effect size were obtained (SMD=0.24, 95% CI 0.08 to 0.40), demonstrating the robustness of the findings.

## Discussion

### Principal Findings

The current meta-analysis found moderate-quality evidence that digital physiotherapy interventions were effective in improving physical function with small effect size (SMD: 0.24, 95% CI 0.13 to 0.35) in individuals with knee OA, compared with standard physiotherapy. Narrative synthesis also showed at least non-inferiority of digital physiotherapy interventions on pain relief. The advantages of digital interventions over conventional therapy have been widely documented.^11 62 63^ Digital interventions can be easily accessed at any time and place, increasing flexibility for patients.^64^ Less transportation cost and time consumption were required, enhancing cost-effectiveness.^40 63^ Interactive features in digital interventions could provide real-time feedback and enhance treatment attractiveness, improving patient adherence.^38 62^

All physiotherapy interventions analysed in the included studies fit into at least one of the four management types stated in the Methods section as recommended by NICE guidelines. Interventions could be either single-component (eg, exercise alone) or multi-component (eg. education and exercise). Therefore, studies adopting more than one treatment modality were also included.

Previous research into digital physiotherapy in improving physical functions for patients with knee OA have been inconsistent. While this study is in agreement with numerous telehealth reviews in improving physical function,^18^,^2062 65^ it differs from recent meta-analyses, suggesting no clinical significance in physical function.^15^,^1722^ This discrepancy could be attributed to the differences in inclusion criteria. Three meta-analyses included psychotherapy, pain coaching and electrical stimulation,^15^,^17^ which were not recommended by national guidelines as evidence-based interventions targeting physical function^66^; one systematic review included five trials that recruited patients with chronic knee pain in addition to knee OA, and this difference in diagnosis could likely affect the pooled results.^22^ When compared with the most recent meta-analysis on digitally delivered exercises on patients with knee or hip OA,^21^ our review largely aligns with its results, indicating significant improvement in physical function. However, this previous study included mixed populations with hip and knee OA and focused on comparing control groups of different traditional management approaches. In contrast, our study focused exclusively on physiotherapy-based digital interventions for only knee OA. We also highlighted the effects of different digital medium and individualised exercise components, which have not been considered by any previous reviews and meta-analyses.

In contrast to physical function, our result accords with the current literature, demonstrating some effects of pain relief with digital interventions. Systematic reviews on digital self-management programmes and educational and exercise-based telehealth interventions consistently showed significant improvement in knee pain.^15^,^1820 65^ Education and self-management were shown to improve illness perceptions and fear-avoidance behaviour, which reduced pain sensitivity^67^; Exercise interventions could increase muscle strength and decrease localised stress on articular cartilage, hence reducing pain.^68^ On the other hand, our results on QoL were mixed, which also reflected the current literature of having limited and inconsistent evidence in evaluating the effectiveness of digital physiotherapy on QoL.^19^,^2160^ In the current study, only 15 out of 25 studies reported QoL. A huge variety of QoL measures, such as AQoL, MSK-HQ, SF-36, subscale of KOOS, were used, making integration and interpretation of results difficult.

Subgroup analyses of digital medium revealed that video-conferencing and app-/web-based physiotherapy yielded statistically significant reduction in pain and improvement in physical functions compared with non-digital physiotherapy. The results matched two recent systematic reviews, illustrating that the use of technologies in web-based programmes and mobile applications was effective to improve pain and functions for patients with knee OA.^22 62^ A possible explanation is that these two digital platforms provided video-based or real-time exercise demonstrations for patients, helping them adjust movement, speed and techniques when performing exercises at home.^22 50^ This resulted in improved accuracy and effectiveness of rehabilitation, which outpatient physiotherapy or paper handouts were unable to provide.^49 50^ However, it is also recognised that there are other expectations in a patient’s journey which may include patient engagement and preference of being connected to a healthcare clinician.^69^ On the contrary, insignificant results were obtained for most telephone-based and text-based physiotherapy for all outcomes, which largely corroborate with previous findings.^26 70^ Many authors speculated that motivational messages and phone call check-ins were too simple and non-specific to create behavioural change,^41 56^ because these two modalities lacked exercise-specific elements.^57^ This prompted a subgroup analysis of individualised exercise components in this study.

Subgroup analysis showed moderate-quality evidence that studies incorporating individualised exercise components were significantly more effective than non-exercise counterparts in improving pain and physical functions, regardless of digital modalities. This result was supported by another systematic review, suggesting that personalised information and exercise for individuals were recommended for digital interventions.^22^ Hinman and colleagues further supported this idea by demonstrating that telephone-delivered individualised exercise advice by physiotherapists improved pain and physical functions.^42^ Individualised therapeutic exercises are strongly recommended by NICE guidelines.^23^ Adjustment in exercises tailored to one’s needs could help limit training plateau and achieve higher levels of strength and functions.^71^ Regular modification and monitoring of exercise programmes provide positive exercise beliefs and self-efficacy, maintaining patients’ motivation.^72^ It is therefore proposed that the inclusion of individualised exercise advice/programmes might be beneficial for successful knee OA digital rehabilitation. Future research should be carried out to evaluate this notion.

Subgroup analysis of length of follow-up indicated that the largest improvement in pain and physical function was short-term (3 months or less), and effect sizes gradually decreased as length of follow-up increased. The result is largely consistent with past reviews, showing that short-term digital interventions yielded the best effectiveness in clinical outcomes, while contradictory results were obtained for long-term follow-up.^18 19 62^ This could be due to the fact that patients usually adhered to exercises better initially, and adherence slowly declined because the attractive features in telerehabilitation might gradually lose their appeal.^62 72^ Patients were also less able to manage symptoms and progress treatment without professional guidance as the length of follow-up increased.^72^ However, the results of subgroup analyses should be interpreted with caution due to moderate to high heterogeneity and multiple comparisons across all three outcome measures.

Although there was not adequate data for meta-analysis, three studies of exercise-based video games were shown to be more effective, or at least non-inferior to traditional exercises in improving pain and physical functions. For example, one study incorporated exergaming where patients needed to perform squats to control the movement of characters in the game.^52^ These games provide enjoyment and real-time visual feedback and motivate patients to practise in an interactive way, enhancing treatment adherence.^43 52^ However, with the current limited evidence, the effect of digital gaming for patients with knee OA remains inconclusive.

### Digital physiotherapy and health inequalities

Beyond clinical effectiveness, digital physiotherapy has the potential to enhance accessibility and equity in musculoskeletal care. Remote delivery models can overcome geographical barriers, reduce travel burden and facilitate continuity of care for individuals with mobility limitations or those living in rural or underserved regions.^73^ This is particularly relevant in healthcare systems with long waiting times or limited physiotherapy capacity. However, digital approaches may inadvertently widen health inequalities if barriers such as low digital literacy, limited access to technology, language barriers or unreliable internet connectivity are not addressed.^74^ Evidence suggests that older adults, individuals with lower socioeconomic status and those for whom English is not a first language are at greater risk of digital exclusion.^75 76^

To maximise equity, clinicians should assess patients’ digital readiness and provide tailored guidance or hybrid (in-person and digital) models where appropriate.^77^ Policymakers should facilitate better communication on the benefits of using digital platforms and prioritise digital inclusion strategies—such as subsidised devices, community-based support for digital literacy and accessible interface design—to ensure that digital physiotherapy complements, rather than replaces, face-to-face care.^69 77^ Raising public awareness around the role of organisations that verify and assess the quality of digital health platforms is also recommended.^69^ Co-designing digital interventions with patients, clinicians and community partners can further enhance acceptability, engagement and real-world impact.^74^

### Strengths and limitations

This study has the following strengths: It is a comprehensive meta-analysis and systematic review including 25 RCTs and a wide range of digital modalities. Unlike previous reviews, we only included “active” physiotherapy interventions, instead of passive control, as comparison groups to emphasise the benefits of digital components. It also updates the evidence pool, as 21 out of 25 RCTs included were published after 2020, most of which were not included in recently published reviews. However, there are several limitations. First, a high ROB was observed in almost half of the trials. Outcome assessment bias due to lack of blinding was a common item in digital physiotherapy, producing biased outcome measurement.^78^ Attrition bias was also common among trials, and a high attrition rate would create a skewed result, especially when participants in the control group might find the intervention ineffective and drop out.^44^ Second, substantial heterogeneity was observed across studies. Large differences in content and delivery modes across intervention and comparison groups, and differences in length of follow-up are likely to have led to the level of heterogeneity observed. As a result, a meta-analysis of pain and QoL could not be performed.^34^ Third, all of the analysed outcome measures on pain, function and QoL were self-reported. Results could have deviated into a biased direction considering that outcome assessment bias was common among the included studies. Lastly, study selection and data extraction were not independently completed by two reviewers, thereby increasing the risk of potential selection bias.

Apart from the above limitations, an important challenge encountered in this review was that over half of the included studies did not report sufficient quantitative data (eg, SD or CIs) to permit inclusion in the meta-analysis, and most corresponding authors did not respond to data requests. This limitation restricted the ability to synthesise all available evidence and reflects a broader issue of incomplete reporting and limited data transparency in digital physiotherapy research. Improving adherence to reporting guidelines such as CONSORT and promoting open data sharing would facilitate more robust meta-analytic evaluation and enhance the reproducibility of future evidence synthesis.

### Conclusion and clinical implications

The current meta-analysis revealed moderate-quality evidence that digital physiotherapy interventions improved physical function statistically significantly compared with non-digital counterparts for individuals with knee OA, but the changes were small to modest. Therefore, it still remains uncertain whether these changes would be considered clinically meaningful. Given its low risk and potential additive value, clinicians may consider digital physiotherapy interventions as an alternative or complementary approach to improve function in patients with knee OA if traditional interventions are not available or accessible to patients.

Qualitative analysis showed at least non-inferiority in pain reduction using digital physiotherapy interventions. The clinical effect on pain and function was best in the short term and decreased gradually as length of follow-up increased. Video-conferencing and app-/web-based physiotherapy interventions appeared to be two of the most effective digital health tools for knee OA rehabilitation. Individualised exercise components could also be incorporated to maximise improvements in pain and function. However, there are still limited high-quality trials investigating the use of digital physiotherapy interventions for clinically supporting patients with knee OA. Future research should minimise the risk of selection, attrition and outcome assessment bias to ensure high-quality trials, investigate the benefits of digital physiotherapy on QoL, and maintain a longer length of follow-up after interventions. Adoption of standardised and valid outcome measures on QoL is highly encouraged to ensure comparability across different trials. Given the limited current preliminary findings, the effect of digital gaming on pain and functions should also be further investigated.

## Supplementary material

10.1136/bmjopen-2025-102887 online supplemental file 1

10.1136/bmjopen-2025-102887 online supplemental file 2

10.1136/bmjopen-2025-102887 online supplemental file 3

10.1136/bmjopen-2025-102887 online supplemental file 4

10.1136/bmjopen-2025-102887 online supplemental file 5

## Data Availability

Data are available upon reasonable request.
