# Supplementary material for: Effectiveness of digital physiotherapy interventions in patients with knee osteoarthritis: a systematic review and meta-analysis of randomised controlled trials
Source: BMJ Open. 2025 Dec 11;15(12):e102887. doi: 10.1136/bmjopen-2025-102887 (PMC12699664; doi:10.1136/bmjopen-2025-102887)
Supplement: online supplemental file 1 [file bmjopen-15-12-s001.docx]

**Appendix I – Detailed Search Strategies and Exclusion of Studies**

**MEDLINE**

Version: OVID MEDLINE (R) ALL, 1946 to February, 2025

Date run: February 19, 2025

| **ID#** | **Searches** | **Results** |
| --- | --- | --- |
| 1 | digital health.mp. | 7454 |
| 2 | telemedicine/ or telerehabilitation/ | 444348 |
| 3 | (teletherapy or virtual therapy or remote therapy).mp. [mp=title, book title, abstract, original title, name of substance word, subject heading word, floating sub-heading word, keyword heading word, organism supplementary concept word, protocol supplementary concept word, rare disease supplementary concept word, unique identifier, synonyms, population supplementary concept word, anatomy supplementary concept word] | 5980 |
| 4 | (telehealth or e-health or ehealth).mp. [mp=title, book title, abstract, original title, name of substance word, subject heading word, floating sub-heading word, keyword heading word, organism supplementary concept word, protocol supplementary concept word, rare disease supplementary concept word, unique identifier, synonyms, population supplementary concept word, anatomy supplementary concept word] | 21948 |
| 5 | technolog*.mp. | 640780 |
| 6 | Mobile Applications/ | 14168 |
| 7 | Internet/ or Internet-Based Intervention/ | 86316 |
| 8 | (Web or website or webpage or web-based intervention).mp. [mp=title, book title, abstract, original title, name of substance word, subject heading word, floating sub-heading word, keyword heading word, organism supplementary concept word, protocol supplementary concept word, rare disease supplementary concept word, unique identifier, synonyms, population supplementary concept word, anatomy supplementary concept word] | 195164 |
| 9 | online therapy.mp. | 194 |
| 10 | Software/ | 131570 |
| 11 | Videotape Recording/ or video.mp. or Video Recording/ or Video-Audio Media/ | 175883 |
| 12 | Social Media/ | 19011 |
| 13 | Text Messaging/ | 4987 |
| 14 | (mobile or mobile phone or telephone or cell phone or smartphone).mp. [mp=title, book title, abstract, original title, name of substance word, subject heading word, floating sub-heading word, keyword heading word, organism supplementary concept word, protocol supplementary concept word, rare disease supplementary concept word, unique identifier, synonyms, population supplementary concept word, anatomy supplementary concept word] | 203504 |
| 15 | Wearable Electronic Devices/ or smart watch.mp. | 10641 |
| 16 | Computers, Handheld/ | 4194 |
| 17 | Osteoarthritis, Knee/ | 30434 |
| 18 | knee OA.mp. | 7038 |
| 19 | randomi*ed controlled trial.mp. | 670436 |
| 20 | 1 or 2 or 3 or 4 or 5 or 6 or 7 or 8 or 9 or 10 or 11 or 12 or 13 or 14 or 15 or 16 | 1347815 |
| 21 | 17 or 18 | 31218 |
| 22 | 19 and 20 and 21 | 365 |
| 23 | limit 22 to yr="2013 - Current" | 290 |

**EMBASE**

Version: EMBASE 1974 to February 18

Interface: OVID

Date run: February 19, 2025

| **ID#** | **Searches** | **Results** |
| --- | --- | --- |
| 1 | telehealth/ | 24291 |
| 2 | telerehabilitation/ | 3587 |
| 3 | teletherapy/ | 1161 |
| 4 | telemedicine/ | 51874 |
| 5 | digital health.mp. | 13199 |
| 6 | technolog*.mp. | 1104785 |
| 7 | mobile application/ | 26225 |
| 8 | internet/ | 129805 |
| 9 | web-based intervention/ | 3919 |
| 10 | software/ | 316118 |
| 11 | videorecording/ | 131986 |
| 12 | social media/ | 62717 |
| 13 | text messaging/ | 9142 |
| 14 | mobile phone/ | 24567 |
| 15 | smartphone/ | 33328 |
| 16 | smart watch/ | 1208 |
| 17 | tablet computer/ | 4249 |
| 18 | computer assisted therapy/ | 4894 |
| 19 | knee osteoarthritis/ | 50581 |
| 20 | knee OA.mp. | 16110 |
| 21 | Randomi?ed controlled trial.mp. | 1181558 |
| 22 | 1 or 2 or 3 or 4 or 5 or 6 or 7 or 8 or 9 or 10 or 11 or 12 or 13 or 14 or 15 or 16 or 17 or 18 | 1754292 |
| 23 | 19 or 20 | 53257 |
| 24 | 21 and 22 and 23 | 495 |
| 25 | Limit 24 to yr=’2013-Current’ | 448 |

**PsycInfo**

Version: APA PsycInfo 1967 to February 2025 Week 1

Interface: OVID

Date run: February 19, 2025

| **ID#** | **Searches** | **Results** |
| --- | --- | --- |
| 1 | digital interventions/ | 2709 |
| 2 | telemedicine/ or exp computer assisted therapy/ or exp electronic health services/ or exp online therapy/ or exp teleconferencing/ or exp teleconsultation/ or exp telerehabilitation/ or exp internet/ | 64448 |
| 3 | technolog*.mp. | 171205 |
| 4 | mobile applications/ or exp computer applications/ or exp mobile technology/ | 97865 |
| 5 | Software.mp. | 43997 |
| 6 | exp video-based interventions/ or exp digital video/ or exp videoconferencing/ | 4708 |
| 7 | social media/ or exp computer mediated communication/ or exp digital media/ or exp online social networks/ | 62153 |
| 8 | text messaging/ or exp electronic communication/ or exp messages/ | 91386 |
| 9 | mobile phones/ or exp mobile devices/ or exp telephone systems/ or exp smartphones/ or exp mobile applications/ or exp "smartphone use"/ | 18774 |
| 10 | wearable devices/ or exp wireless technologies/ | 2076 |
| 11 | tablet computers/ or exp digital gaming/ | 4561 |
| 12 | computers/ or exp computer systems/ or exp computer software/ or exp computer usage/ or exp digital computers/ | 90447 |
| 13 | 1 or 2 or 3 or 4 or 5 or 6 or 7 or 8 or 9 or 10 or 11 or 12 | 364005 |
| 14 | knee osteoarthritis.mp. | 683 |
| 15 | knee OA.mp. | 238 |
| 16 | randomised controlled trial.mp. | 5384 |
| 17 | randomized controlled trials/ or exp randomized clinical trials/ | 1726 |
| 18 | 14 or 15 | 736 |
| 19 | 16 or 17 | 7041 |
| 20 | 13 and 18 and 19 | 0 |

**CINAHL**

Version: CINAHL Plus
Interface: EBSCOhost

Date run: February 19, 2025

| **Search ID#** | **Search Terms** | **Search Options** | **Results** |
| --- | --- | --- | --- |
| S24 | S21 AND S22 AND S23 | **Expanders** - Apply equivalent subjects  **Search modes** - Boolean/Phrase | 0 |
| S23 | S19 OR S20 | **Expanders** - Apply equivalent subjects  **Search modes** - Boolean/Phrase | 16,220 |
| S22 | S1 OR S2 OR S3 OR S4 OR S5 OR S6 OR S7 OR S8 OR S9 OR S10 OR S11 OR S12 OR S13 OR S14 OR S15 OR S16 OR S17 OR S18 | **Expanders** - Apply equivalent subjects  **Search modes** - Boolean/Phrase | 303,590 |
| S21 | "randomised controlled trial or randomized controlled trial or rct" | **Expanders** - Apply equivalent subjects  **Search modes** - SmartText Searching | 12,259 |
| S20 | "knee OA" | **Expanders** - Apply equivalent subjects  **Search modes** - Boolean/Phrase | 14,054 |
| S19 | (MH "Osteoarthritis, Knee") | **Expanders** - Apply equivalent subjects  **Search modes** - Boolean/Phrase | 15,397 |
| S18 | (MH "Therapy, Computer Assisted") OR "computer" | **Expanders** - Apply equivalent subjects  **Search modes** - Boolean/Phrase | 149,723 |
| S17 | (MH "Computers, Portable") OR "tablet computers" | **Expanders** - Apply equivalent subjects  **Search modes** - Boolean/Phrase | 1,838 |
| S16 | "smartwatch or wearable devices" | **Expanders** - Apply equivalent subjects  **Search modes** - SmartText Searching | 135 |
| S15 | "mobile or mobile phone" | **Expanders** - Apply equivalent subjects  **Search modes** - Boolean/Phrase | 10 |
| S14 | (MH "Smartphone") OR (MH "Telephone") OR (MH "Cellular Phone") | **Expanders** - Apply equivalent subjects  **Search modes** - Boolean/Phrase | 25,659 |
| S13 | (MH "Text Messaging") OR (MH "Instant Messaging") OR "text" | **Expanders** - Apply equivalent subjects  **Search modes** - Boolean/Phrase | 43,135 |
| S12 | (MH "Social Media") | **Expanders** - Apply equivalent subjects  **Search modes** - Boolean/Phrase | 24,512 |
| S11 | (MH "Videoconferencing") OR (MH "Video Games") OR "video" | **Expanders** - Apply equivalent subjects  **Search modes** - Boolean/Phrase | 47,596 |
| S10 | (MH "Software") | **Expanders** - Apply equivalent subjects  **Search modes** - Boolean/Phrase | 32,747 |
| S9 | "web or website or webpage or web-based intervention" | **Expanders** - Apply equivalent subjects  **Search modes** - SmartText Searching | 262 |
| S8 | (MH "Internet-Based Intervention") OR (MH "Internet") | **Expanders** - Apply equivalent subjects  **Search modes** - Boolean/Phrase | 56,509 |
| S7 | "mobile applications or apps or mobile apps" | **Expanders** - Apply equivalent subjects  **Search modes** - SmartText Searching | 3,879 |
| S6 | "mobile applications or apps or mobile apps" | **Expanders** - Apply equivalent subjects  **Search modes** - Boolean/Phrase | 0 |
| S5 | "technolog*" OR (MH "Digital Technology") OR (MH "Technology") OR (MH "Assistive Technology") | **Expanders** - Apply equivalent subjects  **Search modes** - Boolean/Phrase | 221,257 |
| S4 | (MH "Telemedicine+") OR (MH "Telehealth+") | **Expanders** - Apply equivalent subjects  **Search modes** - Proximity | 48,023 |
| S3 | "teletherapy or remote therapy or virtual therapy" | **Expanders** - Apply equivalent subjects  **Search modes** - SmartText Searching | 212 |
| S2 | "telerehabilitation or tele-rehabilitation or virtual rehabilitation or remote rehabilitation" | **Expanders** - Apply equivalent subjects  **Search modes** - SmartText Searching | 1,413 |
| S1 | (MH "Digital Health+") | **Expanders** - Apply equivalent subjects  **Search modes** - Boolean/Phrase | 32,055 |

**Web of Science Core Collection**

Date run: February 19, 2025

| **Syntax** | **Results** |
| --- | --- |
| "digital health" or "telerehabilitation" or "virtual rehabilitation" or "remote rehabilitation" or "teletherapy" or "virtual therapy" or "remote therapy" or "telemedicine" or "technolog*" or "mobile application" or "apps" or "application" or "Internet" or "web-based intervention" or "web" or "webpage" or "website" or "online" or "software" or "video" or "videorecordings" or "video conferencing" or "social media" or "text" or "message" or "mobile phone" or "mobile" or "phone" or "telephone" or "smartphone" or "smartwatch" or "tablet computer" or "computer" or "computer-assisted therapy" or "wearable device" (All Fields) AND "Knee osteoarthritis" or "Knee OA" (All Fields) AND "randomi?ed controlled trial" (All Fields) and 2025 or 2024 or 2023 or 2022 or 2013 or 2014 or 2015 or 2021 or 2020 or 2019 or 2018 or 2017 or 2016 (Publication Years) and Article (Document Types) | 532 |

<https://www.webofscience.com/wos/woscc/summary/0f9b0f8a-b14e-44a7-ad67-171be33419d9-014a9b5c93/date-descending/1>

**Scopus**

Date run: February 19,2025

| **Syntax** | **Results** |
| --- | --- |
| ( TITLE ( "knee osteoarthritis" OR "knee OA" ) ) AND ( ( TITLE ( "digital health" OR "telerehabilitation" OR "virtual rehabilitation" OR "remote rehabilitation" OR "teletherapy" OR "virtual therapy" OR "remote therapy" OR "telemedicine" OR technolog* ) ) OR ( TITLE ( "mobile application" OR "apps" OR "application" OR "Internet" OR "web-based intervention" OR "web" OR "webpage" OR "website" OR "online" OR "software" OR "video" OR "videorecording" OR "video conferencing" OR "social media" OR "text" OR "message" ) ) OR ( TITLE ( "mobile phone" OR "mobile" OR "phone" OR "telephone" OR "smartphone" OR "smartwatch" OR "tablet computer" OR "computer" OR "computer-assisted therapy" ) ) ) AND ( TITLE-ABS-KEY ( "randomi?ed controlled trial" ) ) AND PUBYEAR > 2012 | 99 |

**Cochrane Library**

Version: Cochrane Database of Systematic Reviews Issue 1 of 12, January 2024

Date run: February 19, 2025

| **ID** | **Syntax** | **Types** | **Results** |
| --- | --- | --- | --- |
| #1 | MeSH descriptor: [Digital Technology] 1 tree(s) exploded | MeSH | 24 |
| #2 | "digital health" | Limits | 1621 |
| #3 | MeSH descriptor: [Telerehabilitation] explode all trees | MeSH | 377 |
| #4 | MeSH descriptor: [Telemedicine] explode all trees | MeSH | 5150 |
| #5 | MeSH descriptor: [Mobile Applications] explode all trees | MeSH | 2225 |
| #6 | MeSH descriptor: [Internet-Based Intervention] explode all trees | MeSH | 824 |
| #7 | MeSH descriptor: [Software] explode all trees | MeSH | 6392 |
| #8 | MeSH descriptor: [Videoconferencing] explode all trees | MeSH | 352 |
| #9 | MeSH descriptor: [Video Recording] explode all trees | MeSH | 3356 |
| #10 | MeSH descriptor: [Video Games] explode all trees | MeSH | 1260 |
| #11 | MeSH descriptor: [Exergaming] explode all trees | MeSH | 75 |
| #12 | MeSH descriptor: [Social Media] explode all trees | MeSH | 613 |
| #13 | MeSH descriptor: [Text Messaging] explode all trees | MeSH | 1670 |
| #14 | MeSH descriptor: [Cell Phone] explode all trees | MeSH | 3570 |
| #15 | MeSH descriptor: [Smartphone] explode all trees | MeSH | 1216 |
| #16 | Smartwatch | Limits | 292 |
| #17 | MeSH descriptor: [Computers, Handheld] explode all trees | MeSH | 1602 |
| #18 | MeSH descriptor: [Therapy, Computer-Assisted] explode all trees | MeSH | 3145 |
| #19 | #1 OR #2 OR #3 OR #4 OR #5 OR #6 OR #7 OR #8 OR #9 OR #10 OR #11 OR #12 OR #13 OR #14 OR #15 OR #16 OR #17 OR #18 | Limit | 22488 |
| #20 | MeSH descriptor: [Osteoarthritis, Knee] explode all trees | MeSH | 6943 |
| #21 | MeSH descriptor: [Randomized Controlled Trial] explode all trees | MeSH | 34 |
| #22 | #19 AND #20 AND #21 | Limits | 0 |

**Reasons for exclusion of studies**

| **Studies** | **Reasons for exclusion** |
| --- | --- |
| Rini et al., 2015 | Inappropriate intervention group. The intervention was mainly a psychological intervention, which did not involve physiotherapy care. |
| Bennell et al., 2017 | Participants were not diagnosed with osteoarthritis. They were presented with chronic knee pain which could be due to causes other than OA. |
| Skrepnik et al., 2017 | Inappropriate control group. Control group received intra-articular Hylan G-F 20 injections only, which was not part of usual physiotherapy care. |
| O’Brien et al., 2018 | Inappropriate control group. Control group was just on an orthopaedic waiting, which did not involve any kinds of physiotherapy care. |
| Hsu et al., 2021 | Inappropriate control group. Control group was diet control, which did not involve any kinds of physiotherapy care. |
| Harris et al., 2023 | It did not measure any one of the targeted outcome measures. |
| Hunter et al., 2023 | Inappropriate control group. Control group was usual GP care, which was predominantly medical management, but not physiotherapy care. |
| Lee et al., 2023 | Inappropriate control group. Control group was passive, which did not involve any kinds of physiotherapy care. |
| Lorbeer et al., 2023 | Inappropriate intervention group. The intervention was mainly a psychological intervention, which did not involve physiotherapy care. |
| Khazaei et al., 2024 | Inappropriate control group. The control group did not receive any education to a similar extent to the tele-education the intervention group received.  Although both groups received exercise programmes, it was not an entirely fair comparison and did not highlight the use of technology over conventional methods. Rather, the intervention group received extra education and counselling. |
| Dieter, Janssen and Krauss, 2024 | Inappropriate control group. The control group just received usual, passive care. |
| Gillcrist et al., 2024 | Inappropriate control group. The control group received less amount of care compared to the intervention group. Their difference was not only in the digital intervention method – they did not have a social partner to provide encouragement. |
| Mesa-Castrillon, 2024 | Participants were diagnosed with lower back pain, not knee osteoarthritis. |
| Adesola et al., 2024 | It was a Quasi Experimental Study, not an RCT. |
| Dieter et al., 2025 | Inappropriate control group. The control group just received usual, passive care. |
| Li et al., 2025 | Inappropriate control group. The control group just received usual, medical care. |
| Lorbeer et al., 2025 | Inappropriate interventions. The intervention group predominantly included psychological/ behavioural therapy, which did not fit in the NICE guideline criteria of physiotherapy. |
| Moutzouri et al., 2024 | Inappropriate control group. The control group has less frequency of encouragement with no exercise prescription video, making an unfair comparison between the two groups |

**REFERENCES**

1. Rini C, Porter LS, Somers TJ, McKee DC, DeVellis RF, Smith M, Winkel G, Ahern DK, Goldman R, Stiller JL, Mariani C. Automated Internet-based pain coping skills training to manage osteoarthritis pain: a randomized controlled trial. Pain. 2015 May 1;156(5):837-48.

2. Bennell KL, Nelligan R, Dobson F, et al. Effectiveness of an internet-delivered exercise and pain-coping skills training intervention for persons with chronic knee pain: a randomized trial. Annals of internal medicine. 2017 Apr 4;166(7):453-62.

3. Skrepnik N, Spitzer A, Altman R, et al. Assessing the impact of a novel smartphone application compared with standard follow-up on mobility of patients with knee osteoarthritis following treatment with Hylan GF 20: a randomized controlled trial. JMIR mHealth and uHealth. 2017 May 9;5(5):e7179.

4. O'Brien KM, Wiggers J, Williams A, et al. Telephone-based weight loss support for patients with knee osteoarthritis: a pragmatic randomised controlled trial. Osteoarthritis and Cartilage. 2018 Apr 1;26(4):485-94.

5. Hsu YI, Chen YC, Lee CL, et al. Effects of diet control and telemedicine-based resistance exercise intervention on patients with obesity and knee osteoarthritis: a randomized control trial. International Journal of Environmental Research and Public Health. 2021 Jul 21;18(15):7744.

6. Harris A, Hinman RS, Lawford BJ, et al. Cost‐Effectiveness of Telehealth‐Delivered Exercise and Dietary Weight Loss Programs for Knee Osteoarthritis Within a Twelve‐Month Randomized Trial. Arthritis Care & Research. 2023 Jun;75(6):1311-9.

7. Hunter DJ, Bowden JL, Hinman RS, et al. Effectiveness of a new service delivery model for management of knee osteoarthritis in primary care: a cluster randomized controlled trial. Arthritis Care & Research. 2023 Jun;75(6):1320-32.

8. Lee EL, Jang MH, Lee BJ, et al. Home-Based Remote Rehabilitation Leads to Superior Outcomes for Older Women With Knee Osteoarthritis: A Randomized Controlled Trial. Journal of the American Medical Directors Association. 2023 Oct 1;24(10):1555-61.

9. Lorbeer N, Knoll N, Keller J, et al. Enhancing physical activity and reducing symptoms of patients with osteoarthritis of the knee: a randomized controlled trial of the PrevOP-Psychological Adherence Program. BMC Musculoskeletal Disorders. 2023 Jul 4;24(1):550.

10. Reyhaneh K, Faezeh M, Zahra B, et al. Developing an 8‐Week, Tele‐Education Weight Control and Exercise Programme, and Evaluating Its Effects on Weight and Pain Reduction in Patients With Obesity and Knee Osteoarthritis: A Double‐Blinded Randomised Clinical Trial. Musculoskeletal Care. 2024 Aug 9;22(3).

11. Dieter V, Janssen P, Krauss I. Efficacy of the mHealth-Based Exercise Intervention re.flex for Patients With Knee Osteoarthritis: Pilot Randomized Controlled Trial. JMIR Mhealth Uhealth. 2024;12:e54356.

12. Gillcrist RL, Doherty CR, Olave M, et al. A Remote Behaviorally Designed Intervention to Promote Physical Activity in Patients With Knee Osteoarthritis. JCR Journal of Clinical Rheumatology. 2024 Oct 16;30(8):336–9.

13. Mesa-Castrillon CI, Simic M, Ferreira ML, et al. EHealth to empower patients with musculoskeletal pain in rural Australia (EMPoweR) a randomised clinical trial: study protocol. BMC Musculoskeletal Disorders. 2021 Jan 5;22(1).

14. Adesola Ojo Ojoawo, Oluwaseun Odesanya, Kayode Kunuji, et al. Effect of clinic-based and telemonitored home-based intervention on pain intensity, functioning and quality of life in patients with knee osteoarthritis. European Journal of Clinical and Experimental Medicine. 2024 Mar 30;22(1):73–81.

15. Dieter, Valerie and Martus, Peter and Seissler et al. Effectiveness of the Self-Directed m-Health Exercise Intervention Re.flex in Patients with Knee Osteoarthritis: A Randomized Controlled Trial. Available at SSRN: <https://ssrn.com/abstract=5096755> or [http://dx.doi.org/10.2139/ssrn.5096755](https://dx.doi.org/10.2139/ssrn.5096755)

16. Li C, Zhu C, Song K, et al. An evidence-based tailored eHealth patient education tool for patients with knee osteoarthritis: A randomized controlled trial. Digital Health. 2025 Jan 1;11.

17. Lorbeer N, Schwarzer R, Keller J, et al. Volitional processes in changing physical activity: A randomized controlled trial with individuals with knee osteoarthritis. Health Psychology. 2024 Dec 16.

18. Moutzouri M, Koumantakis GA, Hurley M, et al. Effectiveness of a Web-Guided Self-Managed Telerehabilitation Program Enhanced with Outdoor Physical Activity on Physical Function, Physical Activity Levels and Pain in Patients with Knee Osteoarthritis: A Randomized Controlled Trial. Journal of Clinical Medicine. 2024 Jan 1;13(4):934.

‌

‌

‌
