# Supplementary material for: Effectiveness of digital physiotherapy interventions in patients with knee osteoarthritis: a systematic review and meta-analysis of randomised controlled trials
Source: BMJ Open. 2025 Dec 11;15(12):e102887. doi: 10.1136/bmjopen-2025-102887 (PMC12699664; doi:10.1136/bmjopen-2025-102887)
Supplement: online supplemental file 2 [file bmjopen-15-12-s002.docx]

**Appendix II: Table 1 Study characteristics of 25 RCTs.**

| **Table 1** Study characteristics of 25 RCTs | | | | | | | |
| --- | --- | --- | --- | --- | --- | --- | --- |
| Author (year) | Country | Participants (n, male %)  (Intervention (I), Control (C)) | Age (mean ± SD) (I,C) | Content of Interventions | | Length of Follow-up (months) | Outcome measures |
|  |  |  |  | Intervention | Comparison |  |  |
| Bennell  (2017) | Australia | 84 (32.1%),  84 (41.7%) | 62 ± 6.9,  63.4 ± 7.8 | Education, physiotherapy exercise and telephone coaching | Education and physiotherapy without telephone coaching | 18 months | NRS pain, WOMAC function subscale, AQoL II |
| Allen (2018) | United States | 140 (31%)*,  142 (29.6%) | 65.3 ± 11.5,  65.7 ± 10.3 | Internet-based exercise training | Outpatient physiotherapy | 12 months | WOMAC total, pain and function |
| Azma  (2018) | Iran | 27 (NI),  27 (NI) | 55 ± 5.2,  56 ± 5.1 | Home exercise program and monitoring through telerehabilitation | Office-based physiotherapy | 6 months | VAS pain, Persian version of KOOS, WOMAC function  subscale |
| Bartholdy  (2019) | Denmark | 19 (21.1%), 19 (26.3%) | 68 ± 7.3,  62 ± 9.7 | OA exercise  program with text messages | OA exercise program without text messages | 1.5 months (6 weeks) | KOOS pain, ADL, sports/rec and QoL  subscale |
| Baker (2020) | United States | 52 (17.3%),  52 (23.9%) | 65.8 ± 6.6,  64.5 ± 8.3 | Group exercise classes and telephone counselling | Group exercise classes without telephone counselling | 24 months | WOMAC pain and function subscale |
| Bennell (2020) | Australia | 56 (38%),  54 (28%) | 61.7 ± 6.7,  62.9 ± 6.8 | Exercise program and behavioural change text messages | Exercise program without text messages | 6 months | NRS pain, KOOS ADL and sports/rec subscale, AQoL-6D |
| Hinman (2020) | Australia | 87 (36.9%),  88 (37.5%) | 62.4 ± 9.1,  62.5 ± 8.1 | Telephone-delivered exercise advice and support | General MSK helpline about OA  and self-management without exercise-specific advice | 12 months | NRS pain, WOMAC function subscale, AQoL-8D |
| Lin (2020) | Taiwan | 40 (40%),  40 (57.5%) | 55.9 ± 15.8,  58.1 ± 16.9 | Active video game (Whack-a-mole and Archery0 | Therapeutic exercise program | 3 months | WOMAC pain, stiffness and function subscale |
| Allen (2021) | United States | 230 (84.3%),  115 (85.2%) | 59.9 ± 9.9,  60.2 ± 11.1 | Internet-based exercise program with assistance of telephone coaching | OA education materials via mail | 9 months | WOMAC pain, stiffness and function subscale |
| Arfaei Chitklar (2021) | Iran | 32 (0%),  32 (0%) | 57.84 ± 8.63,  58.52 ± 6.33 | Mobile-app-based exercise | Face to face session on OA education and exercise | Not specified | WOMAC pain, stiffness and function subscale, SF-36 |
| Gohir (2021) | United Kingdom | 79 (29.2%),  67 (35.1%) | 65.2 ± 9.7,  68 ± 8.6 | Internet-based exercise program and online education | Exercise and information support as per NICE    Self-management plan | 1.5 months (6 weeks) | NRS Pain, WOMAC function subscale, MSK-HQ |
| Nelligan (2021) | Australia | 103 (41.7%),  103 (35.9%) | 60.3 ± 8.2,  59 ± 8.5 | Web-based exercise program and education with assistance of text message | Web-based general information on knee pain, OA and exercise | 6 months | NRS pain, WOMAC function subscale, KOOS function and QoL subscale, AQoL |
| Rafiq (2021) | Pakistan | 38 (44.7%)*,  38 (44.7%) | 53.97 ± 4.36,  51.73 ± 4.93 | App-based lower limb rehab protocol with reminder messages | App-based lower limb rehab protocol without reminder messages | 3 months | WOMAC pain subscale |
| Alasfour (2022) | Saudi Arabia | 20 (0%),  20 (0%) | 53.65 ± 3.96 ,  55.15 ± 4.64 | App-based exercise program | Paper home exercise programs | 1.5 months (6 weeks) | Arabic NRS pain, Arabic WOMAC (reduced version) |
| Bennell (2022) | Australia | 172 (45.9%)*,  68 (33.8%) | 65.4 ± 8.2,  65.3 ± 8.7 | Videoconferencing exercise program and online education | Online education only | 12 months | NRS pain, WOMAC function subscale, AQoL-8D |
| Lin (2022) | Taiwan | 20 (5%),  18 (11.1%) | 75.6 ± 4.4,  76 ± 5.6 | Computer-aided rowing exercise program | Regular face-to-face exercise program | 3  months (12 weeks) | Chinese version of WOMAC pain and function subscale |
| Mete (2022) | Turkey | 60 (22%) | 59.5 (Median)  (IQR 25/75: 55/64) | Exergaming and conventional physiotherapy (Electrotherapy and exercise program) | Conventional physiotherapy (Electrotherapy and exercise program) | 6 weeks | VAS pain, WOMAC pain, stiffness and function subscale |
| Rafiq (2022) | Malaysia | 38 (44.7%)*,  38 (44.7%) | 54 ± 4.4,  52.6 ± 4.6 | Lower limb strengthening exercise and WhatsApp message reminders | Lower limb strengthening exercise without message | 3 months (12 weeks) | WOMAC total score |
| Aily (2023) | Brazil | 50 (40%),  50 (40%) | 53 ± 9,  55 ± 8 | Circuit training through telerehabilitation | Face-to-face circuit training | 26 weeks | VAS pain, WOMAC function subscale |
| Supe (2023) | India | 35 (11.4%), 35 (5.7%) | 58.34 ± 5.8,  58.51 ± 5.66 | Pain science education telerehabilitation and physiotherapy exercise | Only physiotherapy exercise | 2 weeks | NRS pain |
| Thiengwittayaporn (2023) | Thailand | 44 (14.3%),  45 (7.5%) | 62.2 ± 6.8,  63 ± 9.7 | Mobile app-based exercise | Paper handouts exercise | 1 month (4 weeks) | KOOS pain, ADL sports/rec and QoL subscale |
| Tore (2023) | Turkey | 25 (12.5%),  25 (8.3%) | 55.78 ± 7.24,  55.79 ± 6.76 | Videoconferencing physiotherapy | Conventional exercise handout | 2 months (8 weeks) | KOOS pain, ADLs, sports/rec, QoL subscale, NRS pain |
| Hinman (2024) | Australia | 190 (36%), 182 (28%) | 60.5 ± 8.6,  62.2 ± 8.5 | ZOOM video-conferencing exercise program and education | In-person physiotherapy consultations | 9 months (36 week) | NRS pain, WOMAC, AQoL-6D |
| Tümtürk (2024) | Turkey | 29 (20.7%),  28 (42.9%) | 53.59 ±7.12, 51.50 ± 7.03 | WhatsApp Application (exercise program and education) | Instrictional paper forms (exercise program and education) | 8 weeks | VAS pain, WOMAC, EQ-5D-5L |
| Tedeschi (2024) | Italy | 12 (41%),  9 (35%) | 58.6 ± 17.4, 63.6 ± 11.0 | Video-conferencing (knee joint exercise program) | Outpatient physiotherapy (knee joint exercise program) | 3 months (12 weeks) | NRS pain, WOMAC, SF-36 |

ADL = Activities of daily living; AQoL= Assessment of Quality of Life; AQoL II = Assessment of Quality of Life Mark 2; AQoL-6D = Assessment of Quality of Life 6-dimension instrument; AQoL-8D= Assessment of Quality of Life 8-dimension instrument; EQ-5D-5L = European Quality of Life 5 Dimensions 5 Level version; KOOS = Knee Injury and Osteoarthritis Outcome Score; MSK-HQ = Arthritis Research UK Musculoskeletal Health Questionnaire; NRS = Numerical Rating Scale; VAS = Visual analog scale; WOMAC = Western Ontario and McMaster Universities Osteoarthritis Index.

*There were three groups of participants in these studies, but only the two relevant groups (physiotherapy digital health and physiotherapy without digital component) were selected for this review.
