# Supplementary material for: Effectiveness of digital physiotherapy interventions in patients with knee osteoarthritis: a systematic review and meta-analysis of randomised controlled trials
Source: BMJ Open. 2025 Dec 11;15(12):e102887. doi: 10.1136/bmjopen-2025-102887 (PMC12699664; doi:10.1136/bmjopen-2025-102887)
Supplement: online supplemental file 3 [file bmjopen-15-12-s003.docx]

**Appendix III – Details of study-level results**

| **Table S1** All studies reporting on outcome measures for pain | | | | |
| --- | --- | --- | --- | --- |
| Author (year) | Outcome measure(s) used | Direction of effect, favouring intervention or control? | Statistical significance | Reported effect size |
| Bennell (2017) | NRS pain | No difference | p=0.07 (NS) | Not reported |
| Allen (2018) | WOMAC pain | No difference | p=-0.45 (NS) | Not reported |
| Azma (2018) | VAS pain | No difference | p=0.867 (NS) | Not reported |
| Bartholdy (2019) | KOOS pain | No difference | p=0.64 (NS) | Not reported |
| Baker (2020) | WOMAC pain | No difference | p=0.81 (NS) | Not reported |
| Bennell (2020) | NRS pain | No difference | p=0.59 (NS) | -0.2 |
| Hinman (2020) | NRS pain | Intervention | p=0.44 (NS) | 0.3 |
| Lin (2020) | WOMAC pain | No difference | p=0.085 (NS) | 0.044 |
| Allen (2021) | WOMAC pain | No difference | Not reported | -1.4 |
| Arfaei Chiktlar (2021) | WOMAC pain | Intervention | p=0.0001* | Not reported |
| Gohir (2021) | NRS pain | Intervention | p<0.001* | -0.83 |
| Nelligan (2021) | NRS pain | Intervention | p<0.001* | 1.6 |
| Rafiq (2021) | WOMAC pain | Intervention | p=0.022* | -1.07 |
| Alasfour (2022) | Arabic NRS pain | Intervention | p<0.001* | 2.22 |
| Bennell (2022) | NRS pain | Intervention | p=0.028* | -0.7 |
| Lin (2022) | Nil |  |  |  |
| Mete (2022) | VAS pain and WOMAC pain | Intervention | p=0.048* | Not reported |
| Rafiq (2022) | Nil |  |  |  |
| Aily (2023) | VAS pain | No difference | Not reported | -8 |
| Supe (2023) | NRS pain | Intervention | p<0.0001* | Not reported |
| Thiengwittayaporn (2023) | KOOS pain | No difference | 0.279 (NS) | Not reported |
| Tore (2023) | NRS pain and KOOS pain | Intervention | p<0.001* | -3.83 |
| Hinman (2024) | NRS pain | Intervention | p=8.2x10^-8^* | 0.16 |
| Tümtürk (2024) | VAS pain | No difference | p=0.199 (NS) | Not reported |
| Tedeschi (2024) | NRS pain | No difference | p=0.735 (NS) | Not reported |

KOOS= Knee Injury and Osteoarthritis Outcome score; NS= Statically not significant; NRS = Numerical Rating Scale; VAS = Visual analog scale; WOMAC = Western Ontario and McMaster Universities Osteoarthritis Index.

*p<0.05

| **Table S2** Studies reporting on outcome measures for physical function | | | | |
| --- | --- | --- | --- | --- |
| Author (year) | Outcome measure(s) used | Direction of effect, favouring intervention or control? | Statistical significance | Reported effect size |
| Bennell (2017) | WOMAC function | No difference | p=3.9 (NS) | Not reported |
| Allen (2018) | WOMAC function | No difference | p=0.93 (NS) | Not reported |
| Azma (2018) | WOMAC function | No difference | p=0.959 (NS) | Not reported |
| Bartholdy (2019) | KOOS ADL | No difference | p=0.50 (NS) | Not reported |
| Baker (2020) | WOMAC function | No difference | p=0.84 (NS) | Not reported |
| Bennell (2020) | KOOS ADL | No difference | p=0.95 (NS) | -0.2 |
| Hinman (2020) | WOMAC function | No difference | p=0.097 (NS) | 3.1 |
| Lin (2020) | WOMAC function | No difference | p=0.413 (NS) | 0.026 |
| Allen (2021) | WOMAC function | No difference | Not reported | -4.6 |
| Arfaei Chiktlar (2021) | WOMAC function | Intervention | p=0.0001* | Not reported |
| Gohir (2021) | WOMAC function | Intervention | p=0.02* | -0.60 |
| Nelligan (2021) | WOMAC function and KOOS function | Intervention | p=0.002* | 5.2 |
| Rafiq (2021) | Nil |  |  |  |
| Alasfour (2022) | Arabic WOMAC (reduced version) | Intervention | p<0.001* | 5.11 |
| Bennell (2022) | WOMAC function | Intervention | p=0.004* | -4.4 |
| Lin (2022) | Nil |  |  |  |
| Mete (2022) | WOMAC function | Intervention | p=0.001* | Not reported |
| Rafiq (2022) | Nil |  |  |  |
| Aily (2023) | WOMAC function | No difference | Not reported | -1 |
| Supe (2023) | Nil |  |  |  |
| Thiengwittayaporn (2023) | KOOS ADL | Intervention | p=0.002* | Not reported |
| Tore (2023) | KOOS ADL | Intervention | p<0.001* | 29.54 |
| Hinman (2024) | WOMAC | Intervention | p=7.4x10^-14^* | 1.65 |
| Tümtürk (2024) | WOMAC | No difference | p=0.109 (NS) | Not reported |
| Tedeschi (2024) | WOMAC | Intervention | p=0.019* | Not reported |

ADL = Activities of daily living; KOOS= Knee Injury and Osteoarthritis Outcome score; NS= Statically not significant; WOMAC = Western Ontario and McMaster Universities Osteoarthritis Index.

*p<0.05

| **Table S3** Studies reporting on outcome measures for “quality of life” | | | | |
| --- | --- | --- | --- | --- |
| Author (year) | Outcome measure(s) used | Direction of effect, favouring intervention or control? | Statistical significance | Reported effect size |
| Bennell (2017) | AQoL II | No difference | p=0.0 (NS) | Not reported |
| Allen (2018) | Nil |  |  |  |
| Azma (2018) | Nil |  |  |  |
| Bartholdy (2019) | KOOS QoL | No difference | p=0.95 (NS) | Not reported |
| Baker (2020) | Nil |  |  |  |
| Bennell (2020) | AQoL-6D | No difference | p=0.68 (NS) | -0.01 |
| Hinman (2020) | AQoL-8D | No difference | p=0.85 (NS) | 0.00 |
| Lin (2020) | Nil |  |  |  |
| Allen (2021) | Nil |  |  |  |
| Arfaei Chiktlar (2021) | SF-36 | Intervention | p=0.0001* | Not reported |
| Gohir (2021) | MSK-HQ | No difference | p=0.82 (NS) | -0.3 |
| Nelligan (2021) | KOOS QoL and AQoL | Intervention | p=0.002* | -0.07 |
| Rafiq (2021) | Nil |  |  |  |
| Alasfour (2022) | Nil |  |  |  |
| Bennell (2022) | AQoL-8D | No difference | p=0.112 (NS) | 0.03 |
| Lin (2022) | Nil |  |  |  |
| Mete (2022) | Nil |  |  |  |
| Rafiq (2022) | Nil |  |  |  |
| Aily (2023) | Nil |  |  |  |
| Supe (2023) | Nil |  |  |  |
| Thiengwittayaporn (2023) | KOOS QoL | Intervention | p=0.009* | Not reported |
| Tore (2023) | KOOS QoL | Intervention | p<0.001* | 23.95 |
| Hinman (2024) | AQoL-6D | No difference | p=0.86 (NS) | 0.00 |
| Tümtürk (2024) | EQ-5D-5L | Intervention | p=0.005* | Not reported |
| Tedeschi (2024) | SF-36 | No difference | p=0.766 (NS) |  |

AQoL= Assessment of Quality of Life; AQoL II = Assessment of Quality of Life Mark 2; AQoL-6D = Assessment of Quality of Life 6-dimension instrument; AQoL-8D= Assessment of Quality of Life 8-dimension instrument; EQ-5D-5L = European Quality of Life 5 Dimensions 5 Level version; KOOS QoL = Knee Injury and Osteoarthritis Outcome Score Quality of Life subscale; MSK-HQ = Arthritis Research UK Musculoskeletal Health Questionnaire; NS = Statistically not significant; SF-36 = 36-Item Short Form Survey.

*p<0.05
