# Supplementary material for: Effectiveness of digital physiotherapy interventions in patients with knee osteoarthritis: a systematic review and meta-analysis of randomised controlled trials
Source: BMJ Open. 2025 Dec 11;15(12):e102887. doi: 10.1136/bmjopen-2025-102887 (PMC12699664; doi:10.1136/bmjopen-2025-102887)
Supplement: online supplemental file 4 [file bmjopen-15-12-s004.docx]

**Appendix IV: Table 2 GRADE summary of finding table for 7 patient-reported outcome comparisons.**

| **Table 2** GRADE summary of finding table for 7 patient-reported outcome comparisons | | | | | | |
| --- | --- | --- | --- | --- | --- | --- |
| **Summary of findings:** | | | | | | |
| **Digital physiotherapy interventions compared to standard physiotherapy care for individuals with knee osteoarthritis** | | | | | | |
| **Patient or population:** individuals with knee osteoarthritis  **Setting:** primary care, community, outpatient  **Intervention:** digital physiotherapy interventions  **Comparison:** standard physiotherapy care | | | | | | |
| Outcomes | **Anticipated absolute effects^*^** (95% CI) | | Relative effect (95% CI) | № of participants (studies) | Certainty of the evidence (GRADE) | Comments |
|  | **Risk with standard physiotherapy care** | **Risk with digital physiotherapy interventions** |  |  |  |  |
| Physical Functions (All studies) assessed with: WOMAC or KOOS follow-up: range 1.5 months to 24 months | - | SMD **0.24 higher** (0.13 higher to 0.35 higher) | - | 2407 (16 RCTs) | ⨁⨁⨁◯ Moderate^a^ | Digital physiotherapy interventions likely increases physical functions slightly. |
| Pain (video-conferencing vs. standard physiotherapy care) assessed with: NRS, VAS, WOMAC or KOOS follow-up: range 8 weeks to 12 months | - | SMD **0.53 lower** (1.06 lower to 1.01 lower) | - | 836 (5 RCTs) | ⨁⨁◯◯ Low^b,c^ | Digital physiotherapy interventions of video-conferencing may result in a moderate reduction in pain. |
| Pain (app-based or web-based vs. standard physiotherapy care) assessed with: NRS, VAS, WOMAC or KOOS follow-up: range 6 weeks to 12 months | - | SMD **0.47 lower** (0.70 lower to 0.25 lower) | - | 1035 (6 RCTs) | ⨁⨁⨁◯ Moderate^d^ | App-based or web-based digital physiotherapy interventions likely reduces pain. |
| Pain (digital physiotherapy with exercise component vs. standard physiotherapy care) assessed with: NRS, VAS, WOMAC or KOOS follow-up: range 6 weeks to 12 months | - | SMD **0.43 lower** (0.66 lower to 0.21 lower) | - | 2046 (12 RCTs) | ⨁⨁⨁◯ Moderate^e^ | Digital physiotherapy interventions with an individualised exercise component likely reduces pain. |
| Physical Functions (Video-conferencing vs. standard physiotherapy care) assessed with: WOMAC or KOOS follow-up: range 8 weeks to 12 months | - | SMD **0.32 higher** (0.10 higher to 0.54 higher) | - | 836 (5 RCTs) | ⨁⨁◯◯ Low^f^ | Video-conferencing physiotherapy interventions may increase physical functions. |
| Physical functions (app-based or web-based vs. standard physiotherapy care) assessed with: WOMAC or KOOS follow-up: range 6 weeks to 12 months | - | SMD **0.30 higher** (0.09 higher to 0.50 higher) | - | 1035 (6 RCTs) | ⨁⨁⨁◯ Moderate^g^ | App-based or web-based digital physiotherapy interventions likely increases physical functions. |
| Physical Functions (digital physiotherapy with exercise component vs. standard physiotherapy care) assessed with: WOMAC or KOOS follow-up: range 6 weeks to 12 months | - | SMD **0.30 higher** (0.17 higher to 0.43 higher) | - | 2046 (12 RCTs) | ⨁⨁⨁◯ Moderate^h^ | Digital physiotherapy interventions with an exercise component likely increases physical functions. |
| ***The risk in the intervention group** (and its 95% confidence interval) is based on the assumed risk in the comparison group and the **relative effect** of the intervention (and its 95% CI).  **CI:** confidence interval; **SMD:** standardised mean difference | | | | | | |
| **GRADE Working Group grades of evidence** **High certainty:** we are very confident that the true effect lies close to that of the estimate of the effect. **Moderate certainty:** we are moderately confident in the effect estimate: the true effect is likely to be close to the estimate of the effect, but there is a possibility that it is substantially different. **Low certainty:** our confidence in the effect estimate is limited: the true effect may be substantially different from the estimate of the effect. **Very low certainty:** we have very little confidence in the effect estimate: the true effect is likely to be substantially different from the estimate of effect. | | | | | | |

#### Explanations

a. High risk of attrition bias, and some concerns in measurement of subjective function outcomes, therefore downgrading one level.

b. High risk of bias in deviation from interventions and measurement of outcomes in 2 out of 5 studies, creating very serious effects, downgrading two levels.

c. Only moderate effect in reduction of pain observed in this subgroup, thus not upgrading one level.

d. High risk of attrition bias in 2 out of 6 studies, therefore decided to downgrade one level.

e. High attrition bias in 4 studies, and some concerns for allocation concealment.

f. High risk of bias in deviation from interventions and measurement of outcomes in 2 out of 5 studies, creating very serious effects, downgrading two levels.

g. High risk of attrition bias in 2 out of 6 studies, therefore decided to downgrade one level.

h. High attrition bias in 4 studies, and some concerns for allocation concealment.
