# Supplementary material for: Effectiveness of digital physiotherapy interventions in patients with knee osteoarthritis: a systematic review and meta-analysis of randomised controlled trials
Source: BMJ Open. 2025 Dec 11;15(12):e102887. doi: 10.1136/bmjopen-2025-102887 (PMC12699664; doi:10.1136/bmjopen-2025-102887)

**Appendix V – Forest Plots of All Subgroup analyses**

**Subgroup analyses for digital medium**

Figure 1 – Pain


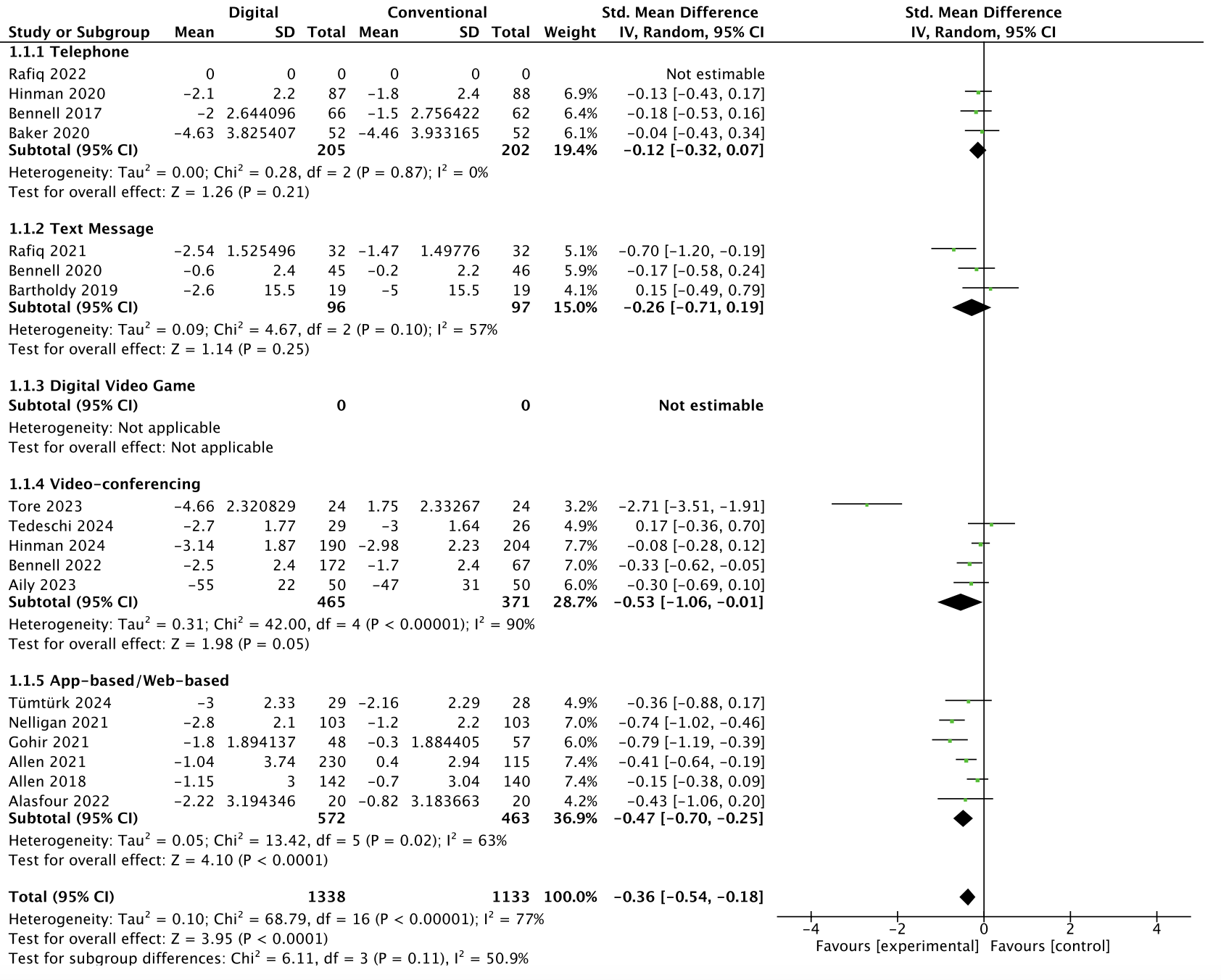


Figure 2 – Physical function


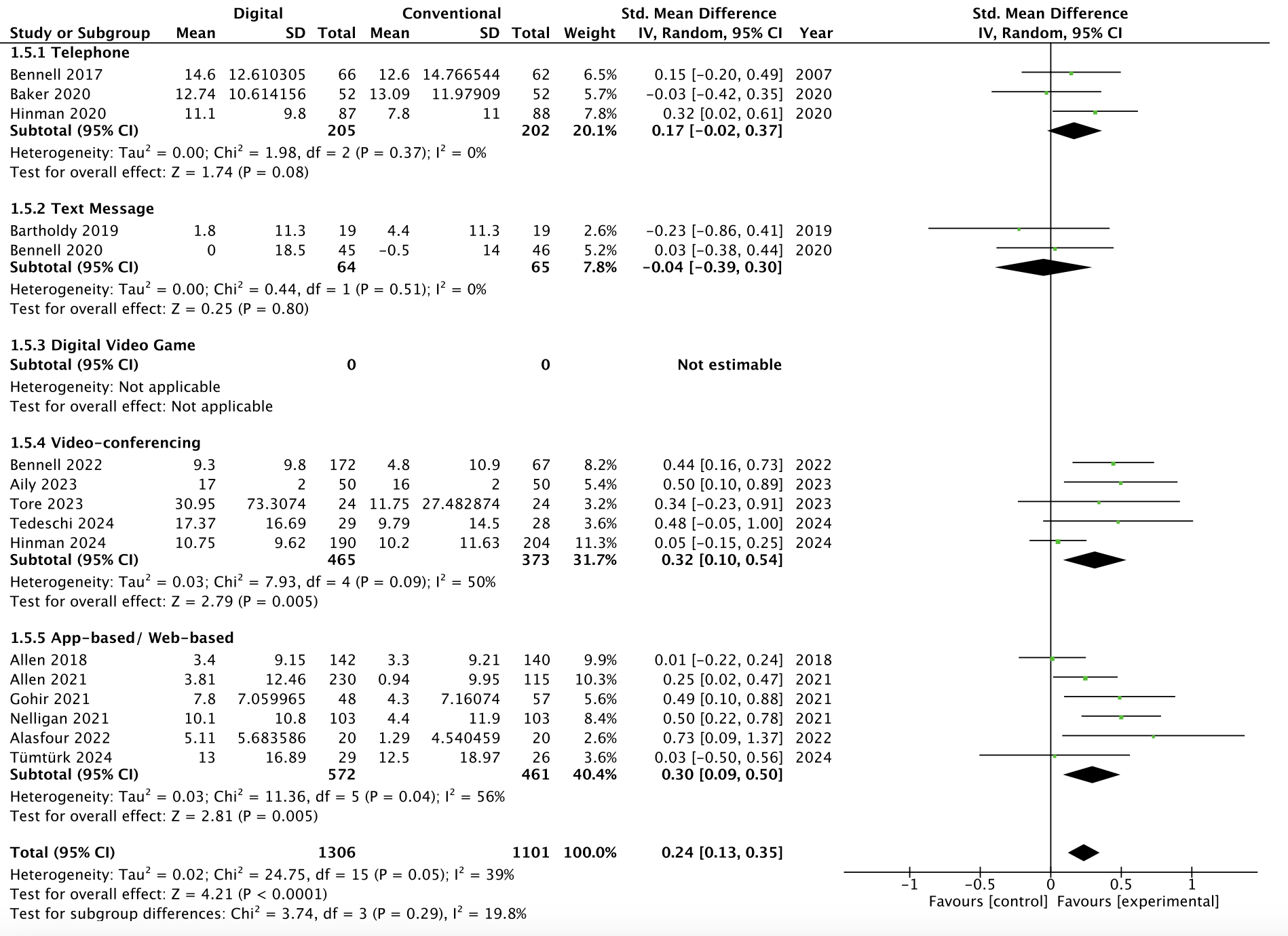


Figure 3 – Quality of Life


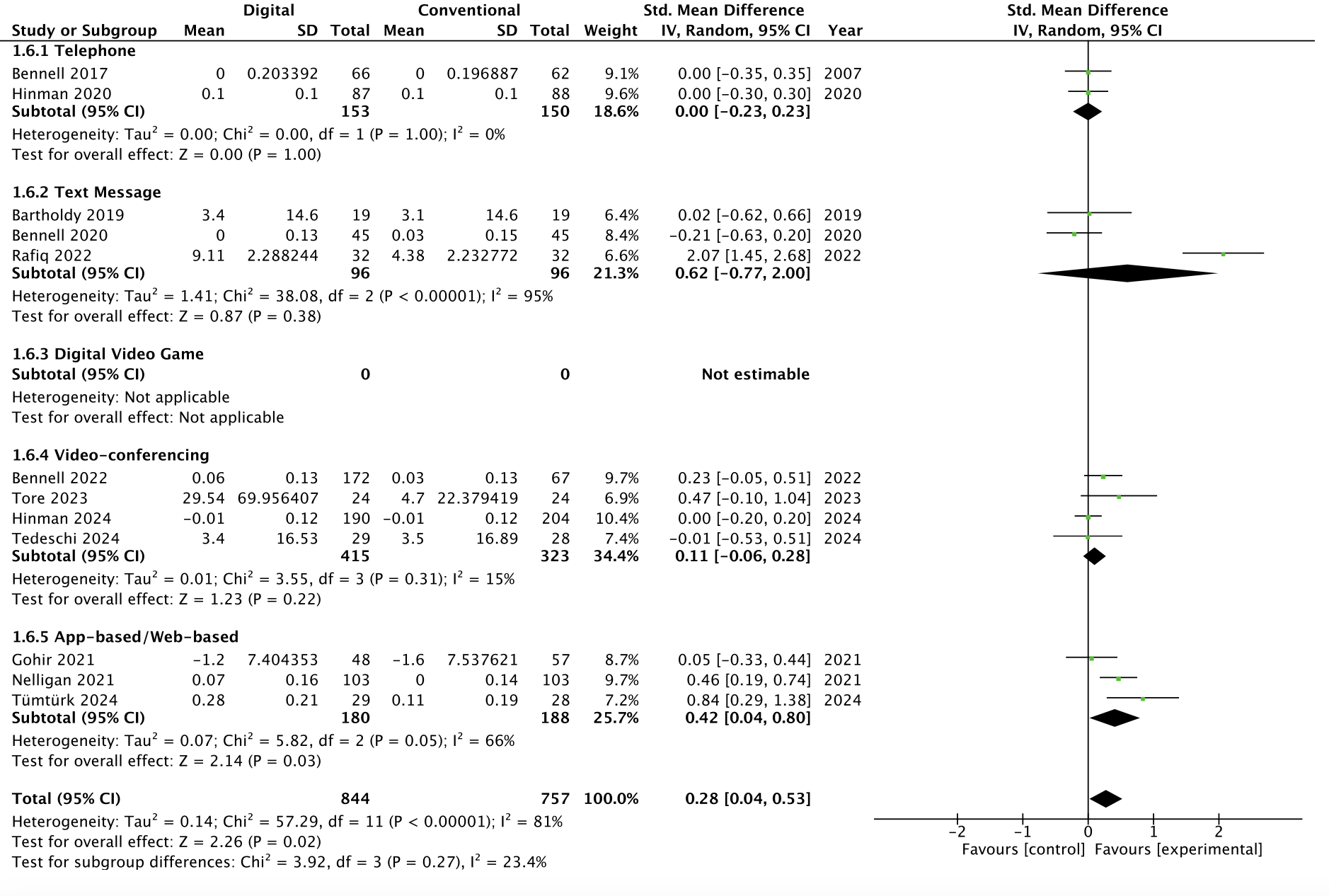


**Subgroup analyses for exercise components**

Figure 4 — pain


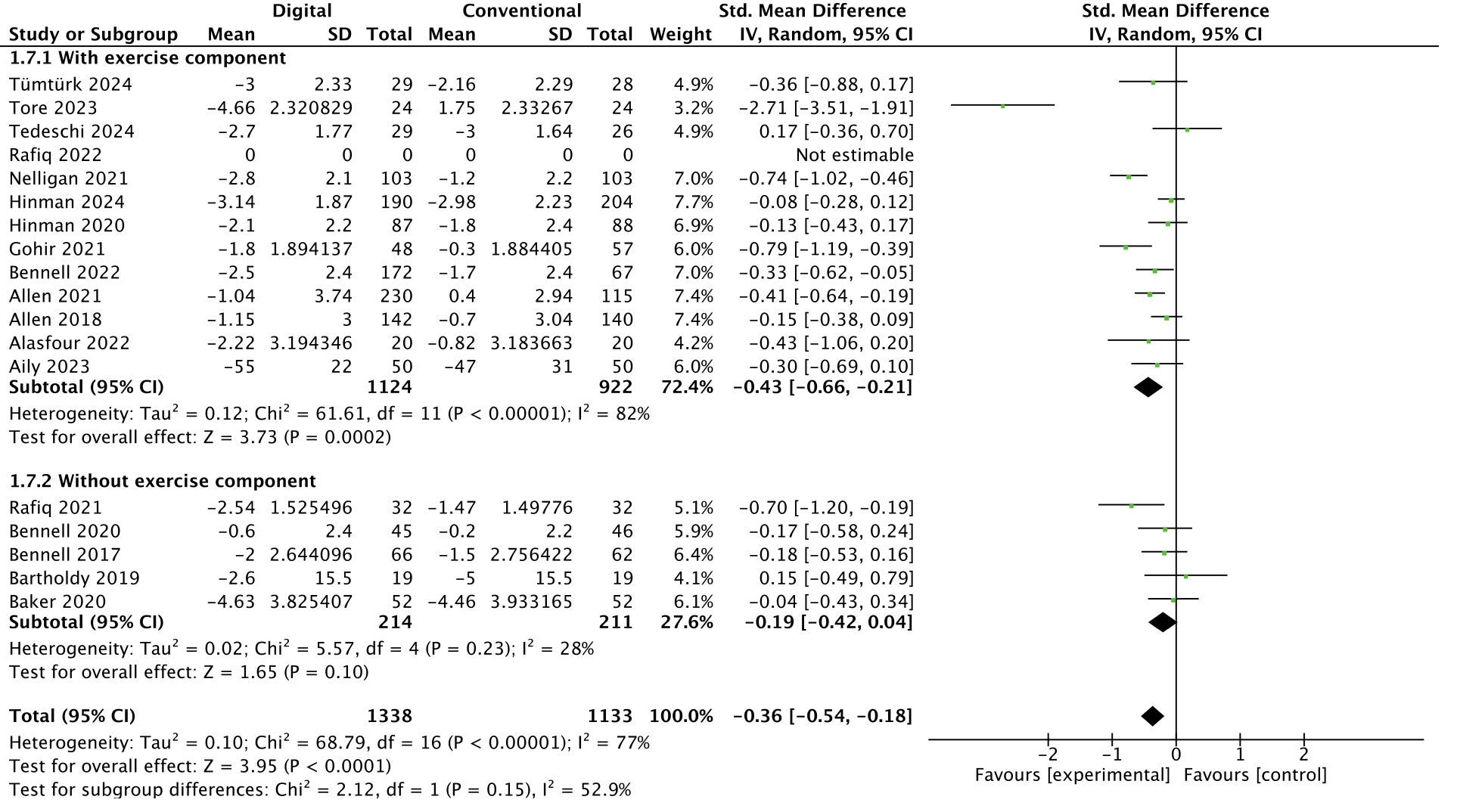


Figure 5 – Physical function


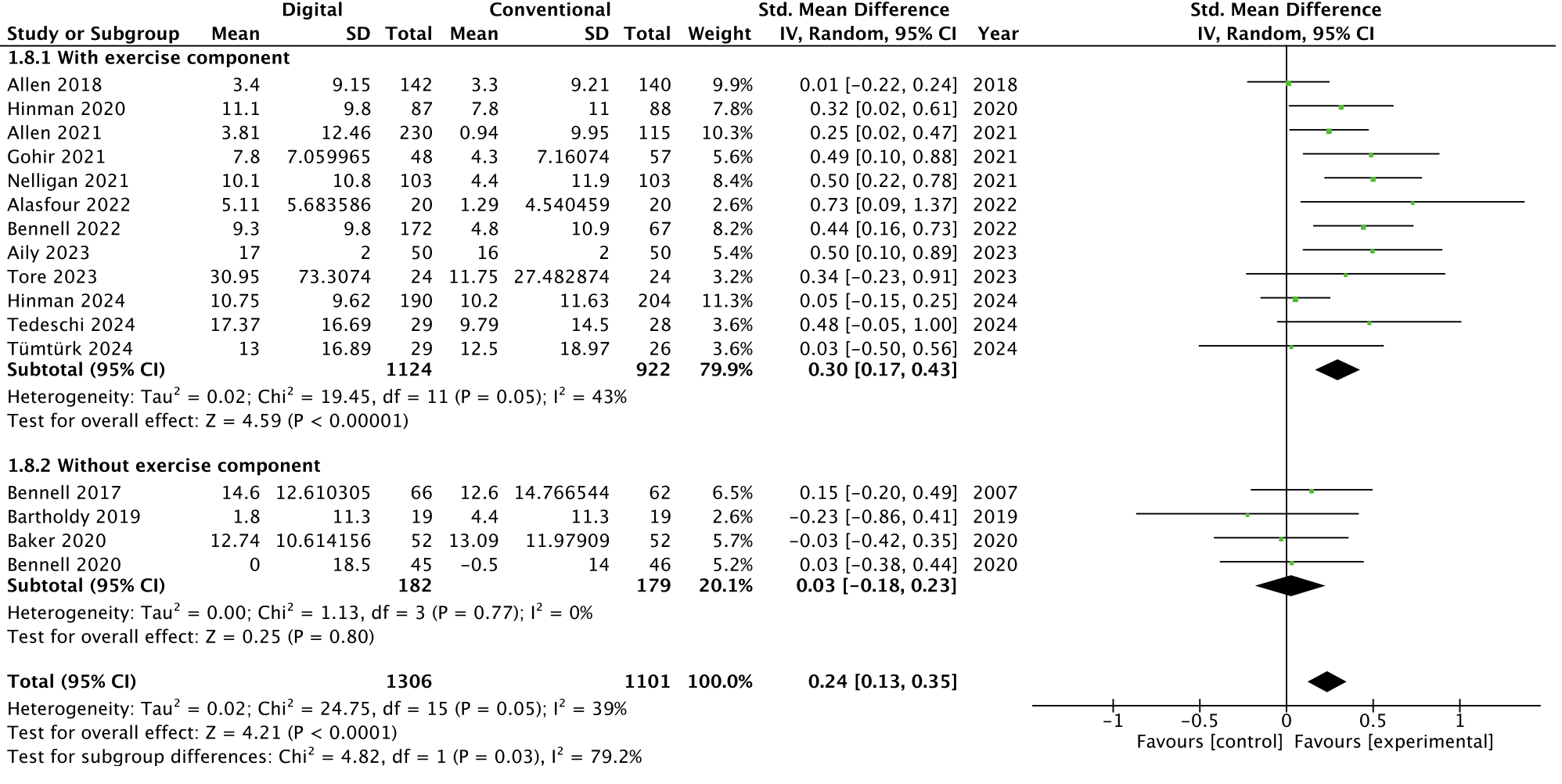


Figure 6 – Quality of Life


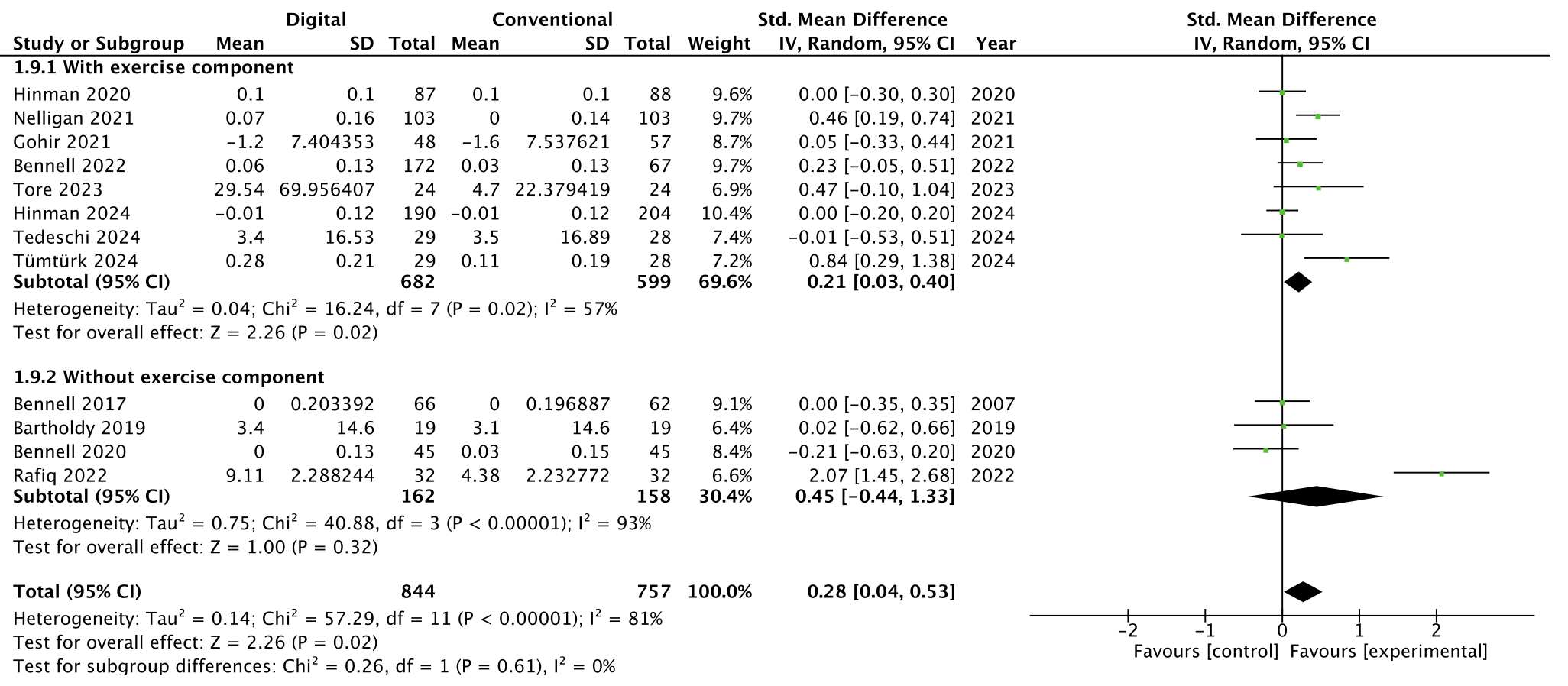


**Subgroup analyses for length of follow-up**

Figure 7 – Pain


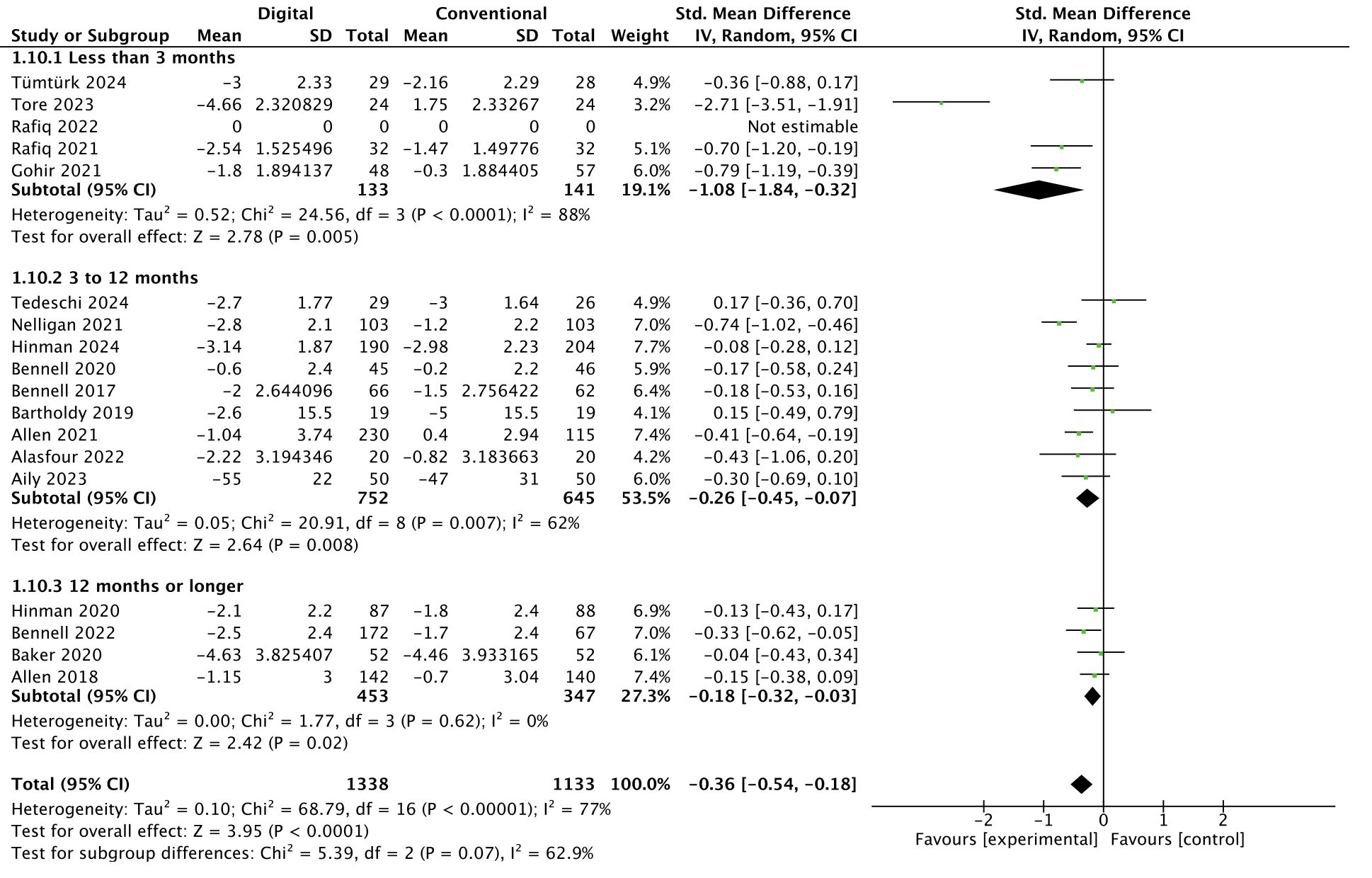


Figure 8 – Physical function


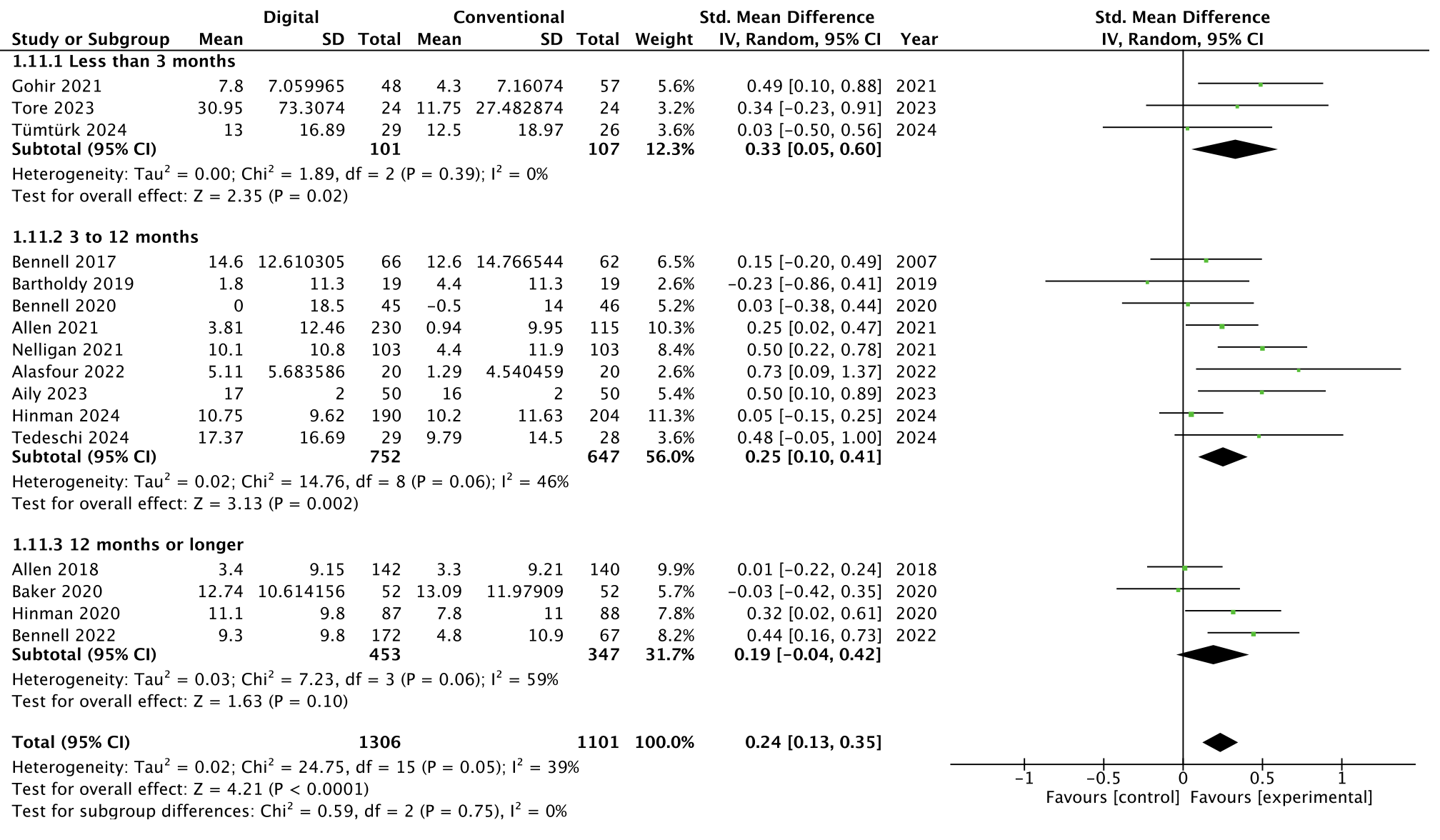


Figure 9 – Quality of Life


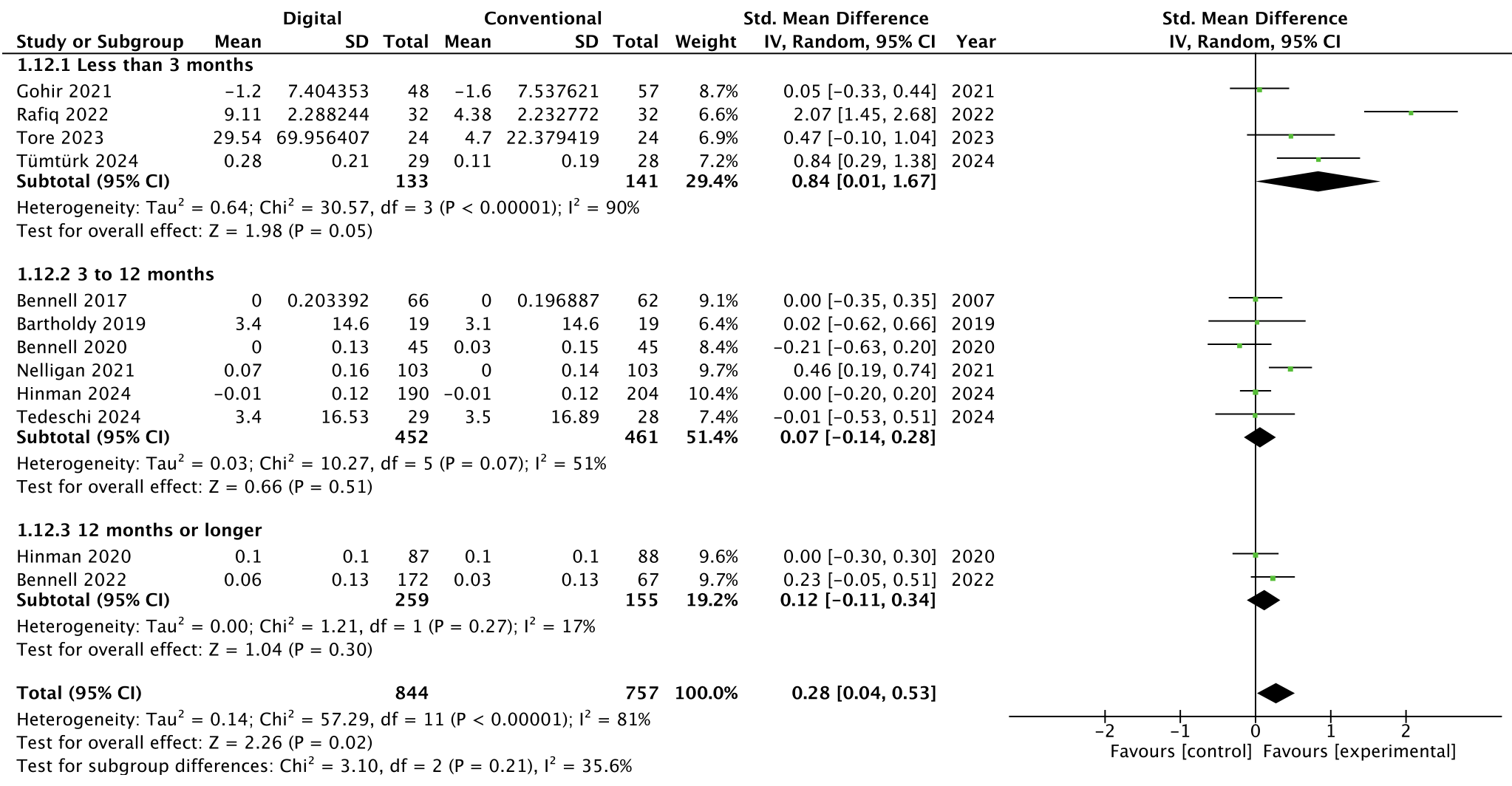

Supplement: online supplemental file 5 [file bmjopen-15-12-s005.docx]
