# Supplementary material for: Effectiveness of digital physiotherapy interventions in patients with knee osteoarthritis: a systematic review and meta-analysis of randomised controlled trials
Source: BMJ Open. 2025 Dec 11;15(12):e102887. doi: 10.1136/bmjopen-2025-102887 (PMC12699664; doi:10.1136/bmjopen-2025-102887)
Supplement: Supplementary file 6 [file bmjopen-2025-102887.reviewer_comments.pdf]

PEER REVIEW HISTORY

BMJ Open publishes all reviews undertaken for accepted manuscripts. Reviewers are asked to complete a checklist review form and are provided with free text boxes to elaborate on their assessment. These free text comments are reproduced below.

ARTICLE DETAILS

Title (Provisional)

The effectiveness of digital physiotherapy interventions in patients with knee osteoarthritis: A systematic review and meta-analysis of randomised controlled trials

Authors

Cheung, Tsz Him Ryan; Elliott, Mark; Stephens, Gareth; Mansfield, Michael

---

VERSION 1 - REVIEW

**Reviewer**	**1**
**Name**	**Resende, Renan**
**Affiliation**	**Universidade Federal de Minas Gerais**
**Date**	**25-May-2025**
**COI**	**None**

General Comments

This is a timely and relevant systematic review and meta-analysis addressing the effectiveness of digital physiotherapy interventions for individuals with knee osteoarthritis. The protocol was prospectively registered (PROSPERO), and the methodology follows PRISMA, GRADE, and Cochrane standards. The inclusion of diverse digital modalities and subgroup analyses adds clinical value. However, some critical methodological issues and interpretative limitations should be addressed before this manuscript can be considered for publication.

Introduction

Improve the transition between the problem statement and the rationale for digital physiotherapy (i.e., from the first to the second paragraph).

Methods

Based on the AMSTAR 2 tool, the methodological quality of this review is rated as critically low, due to one critical weakness and an additional non-critical limitation: study selection was not conducted independently by two reviewers (increasing the risk of selection bias); and data extraction was performed by one reviewer and only checked by a second, rather

than extracted in duplicate. These issues reduce the transparency and reliability of the review process, despite the appropriate use of ROB-2, GRADE, and meta-analytic techniques.

Given that a substantial proportion of the included studies were rated at high risk of bias, did the authors consider conducting a sensitivity analysis excluding these studies? While subgroup analyses were performed based on digital delivery medium, follow-up duration, and inclusion of exercise components, a sensitivity analysis based on methodological quality could provide additional insight into the robustness of the findings.

Results

To enhance transparency and interpretation, consider including structured summary tables with study-level results for all primary outcomes—particularly for pain and quality of life (where meta-analysis was not conducted) and also for physical function (despite the pooled analysis). These tables could report the outcome measures used, direction and significance of effects, and, if available, effect sizes. This would help readers better understand the consistency of findings and contextualize the pooled estimates given the clinical and methodological heterogeneity.

Discussion

Before interpreting the findings, the authors should revisit the use of the term "physiotherapy" throughout the manuscript. While a definition aligned with NICE guidelines is provided in the methods, it would be helpful to reinforce this distinction in the discussion—particularly clarifying whether the interventions analyzed were single-component (e.g., exercise alone) or multi-component approaches. This will improve conceptual clarity and support accurate interpretation.

The authors appropriately acknowledge the heterogeneity in QoL measures. However, they could strengthen this point by suggesting how future research might address it—such as by adopting standardized instruments to facilitate comparability across trials.

**Reviewer**	**2**
**Name**	**Leirós-Rodríguez, Raquel**
**Affiliation**	**Universidad de Leon - Campus de Ponferrada**
**Date**	**30-Jul-2025**
**COI**	**None**

Dear Authors,

This manuscript presents a comprehensive systematic review and meta-analysis evaluating the effectiveness of digital physiotherapy interventions for patients with knee osteoarthritis. The topic is highly relevant and timely given the ongoing digital transformation in healthcare

and the need to evaluate evidence-based digital approaches in musculoskeletal rehabilitation. The study follows PRISMA guidelines, is registered in PROSPERO, and employs robust risk of bias and evidence grading tools.

Overall, the manuscript is well-structured and clearly written, with a thorough description of the search strategy, eligibility criteria, and outcomes. The results contribute to the literature by updating and synthesizing evidence from 25 RCTs, most of which have been published in the last five years.

Major Comments

Scientific Merit and Originality

The review addresses important clinical and methodological gaps regarding the use of digital physiotherapy for knee OA. The inclusion of a broad range of digital modalities and active comparators strengthens the study.

The focus on both pain and function, as well as subgroup analyses by digital modality, exercise individualization, and follow-up length, provides valuable insights.

Methodological Quality

The search strategy is comprehensive and well described. However, please clarify the language restrictions (English only) and justify the exclusion of non-English studies.

Data extraction and risk of bias assessment appear rigorous, but it would be helpful to report the inter-rater agreement (e.g., kappa statistic) between reviewers for study selection and ROB assessment.

The rationale for using a 10-year publication window is reasonable, but further justification could be provided given the rapidly evolving field.

Results and Interpretation

The decision not to meta-analyze pain and QoL due to heterogeneity is justified, but consider providing more detail on the sources of heterogeneity (e.g., intervention content, delivery format, outcome measures).

The effect sizes reported for physical function are small. Please discuss the clinical significance and implications of these findings, particularly for patients and clinicians.

Subgroup analyses are informative, but interpretation should be cautious given multiple comparisons and moderate to high heterogeneity.

The review highlights the lack of high-quality RCTs and frequent methodological limitations (e.g., blinding, attrition), which should be emphasized in the implications for future research.

Presentation and Style

The manuscript is generally well written, but some sections would benefit from minor English editing for conciseness and clarity.

Ensure consistency in terminology, e.g., "digital physiotherapy interventions" vs. "tele-rehabilitation" vs. "eHealth".

Figures and tables are referenced appropriately. Please ensure they are of high quality and that all abbreviations are explained.

The PPIE statement is clear and appropriate.

Limitations

High risk of bias in many included studies and substantial heterogeneity are appropriately acknowledged. However, further discussion on publication bias and possible small-study effects would be beneficial.

Consider highlighting the limitations of self-reported outcome measures and the lack of long-term follow-up in most studies.

References

The references are recent and appropriate. If possible, discuss how your findings compare with those of recent meta-analyses (e.g., Xie et al. 2021, Xiang et al. 2023, Chen et al. 2021).

Specific Comments

Abstract:

Clearly summarizes methods and findings. Consider explicitly stating the main limitation (heterogeneity/high ROB) in the conclusion.

Methods:

Clarify any deviations from PROSPERO registration.

Results:

Provide more granularity on participant characteristics (age, severity of OA, geographic distribution).

Discussion:

Expand on the potential for digital physiotherapy to reduce health inequalities and improve access, but also discuss barriers (digital literacy, access to technology).

Consider practical recommendations for clinicians and policymakers.

Conclusions:

Emphasize the need for higher-quality RCTs and standardized reporting in future studies.

Kind regards

Reviewer 3

**Name**	**Dack, Kyle**
**Affiliation**	**University of Bristol, MRC Integrative Epidemiology Unit**
**Date**	**09-Sep-2025**
**COI**	**None**

Statistical review summary:

This meta-analysis was conducted and reported to a high standard and I have no statistical concerns. While there are some comments below, these are all minor points intended to improve readability.

Abstract:

1. “meta-analysis was not performed for pain and quality of life due to considerable heterogeneity” Suggest rephrasing to “an overall meta-analysis”, because there were other meta-analyses of these outcomes performed (the subgroup analyses).

2. I suggest at some point in the methods or results stating that the comparison group was those receiving standard of care without a digital component.

Introduction:

3. The data regarding the number of knee replacements per year is quite old – from 2011-12. I was able to find an updated statistic from 2021/22, but there may be other, more up to date sources - https://digital.nhs.uk/data-and-information/publications/statistical/patient-reported-outcome-measures-proms/finalised-hip-and-knee-replacement-procedures-april-2021-to-march-2022/patient-profile

Methods:

4. Types of study – the authors reasonably state that the search was limited to the last ten years. However, the actual search dates were Jan 2013 to Feb 2025, which would be twelve years? I suggest explaining the discrepancy or rationale for searching from 2013 instead of 2015.

5. “When heterogeneity was observed among studies (p<.05 or I2>50%)” The approach taken by the authors is reasonable and I have no concerns, but I do suggest the language is modified. Heterogeneity is a scale rather than a binary “present/not present” value, as described in the Cochrane handbook. So I suggest rephrasing to “when moderate or higher heterogeneity was observed”. This change would also align more closely with the subsequent sentence, which already framed heterogeneity in graded terms (“considerable heterogeneity”).

6. “To ensure the robustness of evidence, sensitivity analyses were performed to evaluate the effects of digital interventions by excluding studies with high risk of bias.” The RoB 2 tool

has five domains, how were the ratings of these items collapsed to a single rating per study? I suggest elaborating to clarify.

Results

7. An important issue which was identified during this study is that over 50% of studies lacked adequate data to be included in the meta-analysis, and most failed to respond when contact was made. If there is space it may be useful to note this in the discussion as it is a valuable finding, even if it hindered the primary aims of the study.

8. For each meta-analysis result I recommend reporting the heterogeneity, both in terms of the statistic and interpreting it for nonstatistical audiences, e.g. the first meta-analysis (figure 3) I suggest reporting there was an I2 of 39% which indicates moderate heterogeneity in the true effect size between studies/populations.

9. QoL. The current phrasing “meta-analysis was not conducted for QoL” sounds very absolute and like it also excludes subgroup meta-analyses. I suggest rephrasing so readers are not surprised by later subgroup results.

10. Subgroup analyses: Within each paragraph it would improve readability if these results were reported in the same order as the primary results: physical function, then pain.

11. Subgroup comparisons: The final sentence reports comparisons between the subgroups. For length of followup the comparisons are reported directly after each outcome, and this is easier to follow – I suggest applying to the first paragraph also.

12. Additionally, the reported subgroup results are somewhat confusing because the text first highlights apparent subgroup differences (“Physical function also followed the same trend, with the largest effect size noted in the subgroup of 3-months or less”), but then state that no differences were observed (“However, no subgroup differences were observed (p=0.75, I2=0%)”). This would be clearer if the latter sentence was clarified – e.g. explain what is being compared, and that while the point estimates varied, the 95% CI of individual subgroups overlapped and were therefore not statistically significant.

13. A very minor comment but the final sentence “demonstrating the robustness of the analysis” would be more accurate as “robustness of the findings”.

Discussion

14. Limitations – the first limitation is that high risk of bias was present in many studies. I suggest noting however that after excluding these studies, the meta-analysis of physical function still showed a significant effect.

VERSION 1 - AUTHOR RESPONSE

Reviewer: 1

1. Improve the transition between the problem statement and the rationale for digital physiotherapy (i.e., from the first to the second paragraph).
Response: Thank you for your feedback. We have included a sentence at the end of the first paragraph of Introduction to ensure smoother transition between the two statements.

2. Based on the AMSTAR 2 tool, the methodological quality of this review is rated as critically low, due to one critical weakness and an additional non-critical limitation: study selection was not conducted independently by two reviewers (increasing the risk of selection bias); and data extraction was performed by one reviewer and only checked by a second, rather than extracted in duplicate. These issues reduce the transparency and reliability of the review process, despite the appropriate use of ROB-2, GRADE, and meta-analytic techniques.
Response: Thank you for this valuable feedback. We acknowledge the reviewer's point regarding the methodological assessment based on the AMSTAR 2 tool. In this review, the primary author conducted the initial screening and data extraction, which were independently checked in full by a second reviewer. While this approach ensures verification and quality control, we recognise that it does not fully meet AMSTAR 2's preferred standard of independent duplicate screening and extraction.
We have therefore:
- Explicitly acknowledged this as a methodological limitation in the Discussion section.
- Clarified the process in the Methods section to ensure transparency.
- Noted that all discrepancies identified during checking were resolved through discussion to minimise bias.

These additions increase transparency regarding study selection and data extraction procedures.

3. Given that a substantial proportion of the included studies were rated at high risk of bias, did the authors consider conducting a sensitivity analysis excluding these studies? While subgroup analyses were performed based on digital delivery medium, follow-up duration, and inclusion of exercise components, a sensitivity analysis based on methodological quality could provide additional insight into the robustness of the findings.
Response: We agree that a sensitivity analysis would be valuable to prove better robustness of the findings. In response to this, we have completed a sensitivity analysis on physical function, by excluding 5 studies with high risk of bias, as written in the Results section under "sensitivity analysis — ROB assessment".

4. To enhance transparency and interpretation, consider including structured summary tables with study-level results for all primary outcomes—particularly for pain and quality of life (where meta-analysis was not conducted) and also for physical function (despite the pooled analysis). These tables could report the outcome measures used, direction and significance of effects, and, if available, effect sizes. This would help readers better understand the consistency of findings and contextualize the pooled estimates given the clinical and methodological heterogeneity.

Response: Thank you for this constructive suggestion. We agree that providing structured summary tables would improve transparency and help readers interpret study-level results, especially for outcomes where meta-analysis was not possible due to heterogeneity.
Accordingly, we have:
1. Added three structured summary tables in the Supplementary Materials (Tables S1–S3) summarising study-level results for:
- Pain (Table S1)
- Physical function (Table S2)
- Quality of life (Table S3)

2. Each table now includes:
- Study citation and year
- Outcome measure(s) used
- Direction of effect (favouring intervention or control)
- Statistical significance ($p < 0.05$ or NS)
- Reported effect size (SMD or mean difference, if available)

3. A brief description has been added in the Results section to direct readers to these tables for detailed study-level findings.

These additions enhance transparency and contextualise the pooled results within the broader heterogeneity of included studies.

5. Before interpreting the findings, the authors should revisit the use of the term "physiotherapy" throughout the manuscript. While a definition aligned with NICE guidelines is provided in the methods, it would be helpful to reinforce this distinction in the discussion—particularly clarifying whether the interventions analyzed were single-component (e.g., exercise alone) or multi-component approaches. This will improve conceptual clarity and support accurate interpretation.
Response: Thank you for your valuable feedback. We agree that clarifying the intervention
component would inevitably add clarity to the interpretation of physiotherapy. We have therefore included a paragraph in the Discussion section to specify this point.

6. The authors appropriately acknowledge the heterogeneity in QoL measures. However, they could strengthen this point by suggesting how future research might address it—such as by adopting standardized instruments to facilitate comparability across trials.
Response: We also acknowledge that using standardised instruments in future trials would help strengthen this point. We have added sentence under the "Conclusion and clinical implication" section to illustrate it.

Reviewer 2
1. The review addresses important clinical and methodological gaps regarding the use of digital physiotherapy for knee OA. The inclusion of a broad range of digital modalities and

active comparators strengthens the study.
Response: Nil

2. The focus on both pain and function, as well as subgroup analyses by digital modality, exercise individualization, and follow-up length, provides valuable insights.
Response: Nil

3. The search strategy is comprehensive and well described. However, please clarify the language restrictions (English only) and justify the exclusion of non-English studies.
Response: Thank you for this helpful comment. We confirm that our search was restricted to studies published in English. We have now explicitly stated this in the Methods – Information Sources and Search Strategy section and justified the rationale. Non-English studies were excluded because validated translation services were not available within the project resources. This clarification has been added to enhance transparency.

4. Data extraction and risk of bias assessment appear rigorous, but it would be helpful to report the inter-rater agreement (e.g., kappa statistic) between reviewers for study selection and ROB assessment.
Response: Thank you for this constructive suggestion. We appreciate the importance of reporting inter-rater agreement. In this review, study selection and data extraction were not conducted in duplicate, but rather performed by the primary reviewer and independently checked in full by a second reviewer. As this process involved verification rather than independent parallel assessment, a kappa statistic could not be meaningfully calculated. To enhance transparency, we have clarified this procedure in the "Methods" section and have reported that no unresolved disagreements occurred between reviewers during study selection, data extraction, or risk-of-bias assessment.

5. The rationale for using a 10-year publication window is reasonable, but further justification could be provided given the rapidly evolving field.
Response: Thank you for this thoughtful observation. We fully agree that digital health and physiotherapy technologies are rapidly evolving, and that ongoing developments could influence future evidence syntheses. Our decision to apply a 10-year publication window (January 2013–February 2025) was guided by the aim to capture studies reflecting contemporary digital physiotherapy practice while maintaining a manageable scope and methodological consistency. We believe this timeframe adequately represents the current technological landscape—including mobile applications, web-based platforms, and tele-rehabilitation systems—without the inclusion of older, obsolete formats. We appreciate the reviewer's point and have retained the current timeframe to preserve the focus and comparability of the included trials.

6. The decision not to meta-analyze pain and QoL due to heterogeneity is justified, but consider providing more detail on the sources of heterogeneity (e.g., intervention content, delivery format, outcome measures).
Response: Thank you for the valuable feedback. Sources of heterogeneity have been added to both the analysis of both pain and quality of life outcome measures under "Results" section to further improve the transparency on the reasons for clinical

heterogeneity.

7. The effect sizes reported for physical function are small. Please discuss the clinical significance and implications of these findings, particularly for patients and clinicians.
Response: Thank you for this important reminder. We have added in statements in discussing the clinical significance and potential implications on clinical practice under “Conclusion and clinical implications”.

8. Subgroup analyses are informative, but interpretation should be cautious given multiple comparisons and moderate to high heterogeneity.
Response: Thank you. We also acknowledge this and have highlighted it once again in Discussion.

9. The review highlights the lack of high-quality RCTs and frequent methodological limitations (e.g., blinding, attrition), which should be emphasized in the implications for future research.
Response: We agree, and the original manuscript have already highlighted such notion in the Conclusion section:“ Future research should minimise the risk of selection, attrition and outcome assessment bias to ensure high-quality trials”

10. The manuscript is generally well written, but some sections would benefit from minor English editing for conciseness and clarity.
Response: Thank you for the feedback and we have polished the manuscript to ensure better conciseness and clarity

11. Ensure consistency in terminology, e.g., "digital physiotherapy interventions" vs. "tele-rehabilitation" vs. "eHealth".
Response: Thank you for the constructive feedback. we decide to adhere to the term “digital physiotherapy interventions”, as referenced to the WHO documents on digital interventions. Tele-rehab and eHealth may not comprehensively represent all sort of digital medium as considered in this article.

12. Figures and tables are referenced appropriately. Please ensure they are of high quality and that all abbreviations are explained.
Response: Thank you – we have enhanced the quality of Figures and tables where required, and all abbreviations are explained under the table.

13. The PPIE statement is clear and appropriate.
Response: Nil

14. High risk of bias in many included studies and substantial heterogeneity are appropriately acknowledged. However, further discussion on publication bias and possible small-study effects would be beneficial.
Response: Thank you for this helpful comment. We agree that a brief discussion of potential publication bias and small-study effects would strengthen the manuscript. Although the funnel plot for physical function outcomes did not indicate visual asymmetry (suggesting no clear evidence of publication bias), we recognise that small-

study effects cannot be ruled out, particularly given the modest sample sizes and positive findings in several included RCTs. We have now added a paragraph to the Discussion acknowledging these issues and their potential influence on the pooled estimates

15. Consider highlighting the limitations of self-reported outcome measures and the lack of long-term follow-up in most studies.
Response: Thank you for the great feedback. We have included the suggestions into the "Strength and Limitations" section to further highlight the study's limitations.

16. The references are recent and appropriate. If possible, discuss how your findings compare with those of recent meta-analyses (e.g., Xie et al. 2021, Xiang et al. 2023, Chen et al. 2021).
Response: Thank you for your comments. We also agree that comparison with recent meta-analyses is essential for discussion. We have discussed in the 2nd paragraph of the original manuscript, comparing the current study with recent meta-analyses, and suggested the reason for the discrepancies.

17. Clearly summarizes methods and findings. Consider explicitly stating the main limitation (heterogeneity/high ROB) in the conclusion.
Response: Thank you for the comment. We have stated the above limitations in the conclusion of abstract.

18. Clarify any deviations from PROSPERO registration.
Response: We have clarified that we strictly adhere to PROSPERO registration guidance.

19. Provide more granularity on participant characteristics (age, severity of OA, geographic distribution).
Response: Thank you for this constructive feedback. We acknowledge that participant characteristics are important factors to consider. We have included the age and the geographical location of the participants for all studies in Table 1. With regard to selection criteria, we decided to include all severity of OA for this review as our focus would be on the effect of different digital platforms or intervention components. However, we do acknowledge that the severity of OA could be one of the factors to investigate in future reviews and meta-analyses.

20. Expand on the potential for digital physiotherapy to reduce health inequalities and improve access, but also discuss barriers (digital literacy, access to technology). Consider practical recommendations for clinicians and policymakers.
Response:
Thank you for this excellent suggestion. We agree that the equity and accessibility implications of digital physiotherapy warrant further discussion. We have now expanded the Discussion to highlight how digital physiotherapy may improve access to care—particularly for individuals in remote or underserved areas—and to acknowledge barriers such as digital literacy, access to devices or stable internet, and socioeconomic disparities.

In addition, we have added practical recommendations for clinicians and policymakers, emphasising the need for digital inclusion strategies, patient training, and co-design approaches that address varying levels of digital readiness. These additions strengthen the translational relevance of the review.

21. Emphasize the need for higher-quality RCTs and standardized reporting in future studies.
Response: We have emphasised the need for using standardised tests and outcome measures in the conclusion section.

Reviewer 3

1. “meta-analysis was not performed for pain and quality of life due to considerable heterogeneity” Suggest rephrasing to “an overall meta-analysis”, because there were other meta-analyses of these outcomes performed (the subgroup analyses).
Response: Thank you for the suggestion. This was rephrased in the abstract.

2. I suggest at some point in the methods or results stating that the comparison group was those receiving standard of care without a digital component.
Response: We agree with this and it has already been stated in the Methods section:
“Standard physiotherapy care (i.e. any one of the components listed above from (1) to (4) in NICE guideline) with no or insignificant components of digital health would be considered.

3. The data regarding the number of knee replacements per year is quite old – from 2011-12. I was able to find an updated statistic from 2021/22, but there may be other, more up to date sources - https://digital.nhs.uk/data-and-information/publications/statistical/patient-reported-outcome-measures-proms/finalised-hip-and-knee-replacement-procedures-april-2021-to-march-2022/patient-profile
Response: Thank you for the great help with the updated statistics. We have now updated the number of knee replacements accordingly.

4. Types of study – the authors reasonably state that the search was limited to the last ten years. However, the actual search dates were Jan 2013 to Feb 2025, which would be twelve years? I suggest explaining the discrepancy or rationale for searching from 2013 instead of 2015.
Response:
Thank you for this helpful observation. The review was originally designed and conducted as part of a dissertation project in 2023, with an intended 10-year search window (January 2013 – December 2023). Before submission to BMJ Open, the search was updated in February 2025 to capture newly published trials and maintain the review’s currency. This resulted in an extended range (2013–2025), although the core rationale of focusing on approximately the past decade remains unchanged. We have now clarified this in the Methods – Information Sources and Search Strategy section to avoid confusion.

5. “When heterogeneity was observed among studies ($p<.05$ or $I2>50\%$)” The approach taken by the authors is reasonable and I have no concerns, but I do suggest the language is modified. Heterogeneity is a scale rather than a binary “present/not present” value, as described in the Cochrane handbook. So I suggest rephrasing to “when moderate or higher heterogeneity was observed”. This change would also align more closely with the subsequent sentence, which already framed heterogeneity in graded terms (“considerable heterogeneity”).
Response: Thank you for your thoughtful suggestion. We agree and would edit that accordingly.

6. “To ensure the robustness of evidence, sensitivity analyses were performed to evaluate the effects of digital interventions by excluding studies with high risk of bias.” The RoB 2 tool has five domains, how were the ratings of these items collapsed to a single rating per study? I suggest elaborating to clarify.
Response: Thank you for this helpful comment. We agree that clarification is needed on how the domain-level RoB 2 assessments were synthesised into an overall study-level rating.

In this review, the overall risk of bias for each study was determined according to the Cochrane RoB 2 guidance (Sterne et al., 2019): Studies were rated “low risk of bias” if all five domains were judged “low risk”. Studies were rated “some concerns” if at least one domain raised “some concerns” but none were “high risk”. Studies were rated “high risk of bias” if any single domain was judged “high risk” or if multiple domains raised “some concerns”. This overall judgment was used to conduct the sensitivity analysis, where studies rated “high risk of bias” were excluded to test the robustness of pooled results.

We have added this explanation to the Methods – Risk of Bias and Certainty Assessment section for transparency.

7. An important issue which was identified during this study is that over 50% of studies lacked adequate data to be included in the meta-analysis, and most failed to respond when contact was made. If there is space it may be useful to note this in the discussion as it is a valuable finding, even if it hindered the primary aims of the study.
Response: Thank you for this insightful suggestion. We agree that the limited data availability and low author response rate represent an important observation with implications for transparency and reproducibility in this field. We have now added a paragraph in the Discussion acknowledging that more than half of the included studies did not provide sufficient numerical data for inclusion in the meta-analysis, despite attempts to contact corresponding authors. We highlight this as an important barrier to evidence synthesis and an area where future research reporting can be improved through adherence to CONSORT and open data practices.

8. For each meta-analysis result I recommend reporting the heterogeneity, both in terms of the statistic and interpreting it for nonstatistical audiences, e.g. the first meta-analysis (figure 3) I suggest reporting there was an I2 of 39% which indicates moderate heterogeneity in the true effect size between studies/populations.

Response: Thank you for this helpful recommendation. We agree that explicitly reporting heterogeneity statistics and interpreting them for a broader readership improves transparency and interpretability. We have now added the I^2 statistic for each meta-analysis result and provided brief, plain-language interpretations (e.g. "I^2 = 39%, indicating moderate heterogeneity"). These have been incorporated in the *Results* section and referenced in the *Discussion* to clarify variability across studies.

9. QoL. The current phrasing "meta-analysis was not conducted for QoL" sounds very absolute and like it also excludes subgroup meta-analyses. I suggest rephrasing so readers are not surprised by later subgroup results.
Response: Thank you for this constructive feedback. We have edited to "*An overall meta-analysis was not indicated for pain/ QoL*" in both paragraphs.

10. Subgroup analyses: Within each paragraph it would improve readability if these results were reported in the same order as the primary results: physical function, then pain.
Response: Thank you for this great advice. We absolutely agree and have changed the structure of each paragraph in subgroup analyses.

11. Subgroup comparisons: The final sentence reports comparisons between the subgroups. For length of followup the comparisons are reported directly after each outcome, and this is easier to follow – I suggest applying to the first paragraph also.
Response: Thank you for your advice. We have edited the paragraphs for subgroup analyses accordingly to allow readers to follow more easily.

12. Additionally, the reported subgroup results are somewhat confusing because the text first highlights apparent subgroup differences ("Physical function also followed the same trend, with the largest effect size noted in the subgroup of 3-months or less"), but then state that no differences were observed ("However, no subgroup differences were observed (p=0.75, I2=0%)"). This would be clearer if the latter sentence was clarified – e.g. explain what is being compared, and that while the point estimates varied, the 95% CI of individual subgroups overlapped and were therefore not statistically significant.
Response: We have taken this into consideration and re-structured this paragraph. We have now clearly compared the effect sizes of different subgroups in all outcome measures. Thank you.

13. A very minor comment but the final sentence "demonstrating the robustness of the analysis" would be more accurate as "robustness of the findings".
Response: Thank you for the suggestion. We have edited accordingly.

14. Limitations – the first limitation is that high risk of bias was present in many studies. I suggest noting however that after excluding these studies, the meta-analysis of physical function still showed a significant effect.
Response: In terms of statistical analysis, it did show a significant effect after excluding these high ROB studies. However, these were just the studies included in the meta-analysis of physical function. There were studies with high risk of bias that were not included in meta-analysis of physical function, and these studies would definitely

undermine the overall interpretation of the qualitative results of this systematic review. Thus, we do think it is still one of the limitations of our study.
